# Transcriptional delineation of polysaccharide utilization loci in the human gut commensal *Segatella copri* DSM18205 and co-culture with exemplar *Bacteroides* species on dietary plant glycans

**DOI:** 10.1128/aem.01759-24

**Published:** 2024-12-05

**Authors:** Deepesh Panwar, Jonathon Briggs, Alexander S. C. Fraser, William A. Stewart, Harry Brumer

**Affiliations:** 1 Michael Smith Laboratories, The University of British Columbia 98687 , Vancouver, British Columbia, Canada; 2 Department of Biochemistry and Molecular Biology, The University of British Columbia 175086 , Vancouver, British Columbia, Canada; 3 Department of Chemistry, The University of British Columbia 428105 , Vancouver, British Columbia, Canada; 4 Department of Botany, The University of British Columbia 98685 , Vancouver, British Columbia, Canada; University of Illinois Urbana-Champaign, Urbana, Illinois, USA

**Keywords:** *Segatella*, *Prevotella*, human gut microbiota, dietary fiber, plant cell wall, carbohydrate-active enzymes, polysaccharide utilization locus, *Bacteroides*, *Bacteroidaceae*, *Prevotellaceae*

## Abstract

There is growing interest in members of the genus *Segatella* (family *Prevotellaceae*) as members of a well-balanced human gut microbiota (HGM). *Segatella* are particularly associated with the consumption of a diet rich in plant polysaccharides comprising dietary fiber. However, understanding of the molecular basis of complex carbohydrate utilization in *Segatella* species is currently incomplete. Here, we used RNA sequencing (RNA-seq) of the type strain *Segatella copri* DSM 18205 (previously *Prevotella copri* CB7) to define precisely individual polysaccharide utilization loci (PULs) and associated carbohydrate-active enzymes (CAZymes) that are implicated in the catabolism of common fruit, vegetable, and grain polysaccharides (*viz*. mixed-linkage β-glucans, xyloglucans, xylans, pectins, and inulin). Although many commonalities were observed, several of these systems exhibited significant compositional and organizational differences *vis-à-vis* homologs in the better-studied *Bacteroides* (sister family *Bacteroidaceae*), which predominate in post-industrial HGM. Growth on β-mannans, β(1, 3)-galactans, and microbial β(1, 3)-glucans was not observed, due to an apparent lack of cognate PULs. Most notably, *S. copri* is unable to grow on starch, due to an incomplete starch utilization system (Sus). Subsequent transcriptional profiling of bellwether Ton-B-dependent transporter-encoding genes revealed that PUL upregulation is rapid and general upon transfer from glucose to plant polysaccharides, reflective of de-repression enabling substrate sensing. Distinct from previous observations of *Bacteroides* species, we were unable to observe clearly delineated substrate prioritization on a polysaccharide mixture designed to mimic *in vitro* diverse plant cell wall digesta. Finally, co-culture experiments generally indicated stable co-existence and lack of exclusive competition between *S. copri* and representative HGM *Bacteroides* species (*Bacteroides thetaiotaomicron* and *Bacteroides ovatus*) on individual polysaccharides, except in cases where corresponding PULs were obviously lacking.

**IMPORTANCE:**

There is currently a great level of interest in improving the composition and function of the human gut microbiota (HGM) to improve health. The bacterium *Segatella copri* is prevalent in people who eat plant-rich diets and is therefore associated with a healthy lifestyle. On one hand, our study reveals the specific molecular systems that enable *S. copri* to proliferate on individual plant polysaccharides. On the other, a growing body of data suggests that the inability of *S. copri* to grow on starch and animal glycans, which dominate in post-industrial diets, as well as host mucin, contributes strongly to its displacement from the HGM by *Bacteroides* species, in the absence of direct antagonism.

## INTRODUCTION

The human gut microbiota (HGM) is widely recognized as a major contributor to our nutrition and overall health (1). Although the definition of what constitutes a “healthy” HGM remains elusive, in part because of the compositional and metabolic complexity of this ecosystem, diet is a key factor influencing HGM diversity and its impact on the host (2–7). Many autochthonous taxa in the HGM have evolved to utilize specifically the complex carbohydrates comprising dietary fiber that we are otherwise unable to digest (8). In turn, the HGM provides metabolites, including short-chain fatty acids (SCFA), vitamins, etc., in a symbiotic relationship, and also constitutes an exclusionary barrier to enteropathogens (9). Urbanization and industrialization have introduced major changes in human lifestyles, including a shift away from complex carbohydrate-rich diets, which significantly impacts the composition of the HGM and, thus, its molecular functions (4, 10, 11). Indeed, changes in HGM composition, such as those that occur through the consumption of processed foods high in protein, fat, and readily accessible starch, have been implicated in a range of metabolic, inflammatory, and neurological diseases (4, 5, 7, 12).

Bacteria, which constitute the bulk of the HGM, broadly represent two major phyla: the Gram-positive Bacillota (formerly Firmicutes [13]) and the Gram-negative Bacteriodota (formerly Bacteroidetes [13]) (for a detailed discussion of HGM composition, including other taxa, see references [5, 10]). Within the Bacteriodota, members of the genus *Bacteroides* (family *Bacteroidaceae*) have received the greatest attention regarding their microbiology, (meta)genomics, genetics, and biochemistry (14–16). This is likely a consequence of an early focus on North American and European HGM (17, 18). However, recent metagenomics of a broader diversity of individuals from non-industrialized populations, as well as post-industrial vegetarians and vegans, has revealed that Bacteriodota from the genus *Segatella* (formerly *Prevotella* [19]) predominate in people who regularly eat plant-rich diets (20–25) (reviewed in references [4, 5, 26]). Additionally, analysis of ancient stool samples, immigrants, and gnotobiotic mice suggests “Westernization” of diets is associated with loss of *Segatella* (22, 27, 28). *Segatella* are also associated with improved glucose metabolism (29) and recovery from malnourishment (30–32) through bespoke dietary interventions. These observations are intriguing, given the correlation of plant-rich diets with better health *vis-à-vis* post-industrial diets.

Compared with the *Bacteroides* (14), functional studies of the *Segatella* in the HGM are in their infancy, although individual species have been long-known as members of the human oral microbiota, and the gut microbiota of ruminant and monogastric animals (26, 33, 34). The type-strain *Segatella copri* DSM 18205 (*Sc*DSM18205, previously *Prevotella copri* CB7), was first isolated from human feces in 2007 and has served as an exemplar of the genus in the HGM (35). Very recently, large-scale (meta)genomic analyses have motivated the delineation and renaming of former *Prevotella* spp. into distinct genera (19), and greatly expanded the number of strains included in the *S. copri* species complex (36). For comparative purposes, it is useful to note that these taxa all belong to the Family *Prevotellaceae*, which is sister to the *Bacteroidaceae* (e.g.*, Bacteroides* spp.) in the Order Bacteroidales.

Like the sister genus *Bacteroides*, *Segatella* encode complex systems of carbohydrate-active enzymes (CAZymes), glycan-binding proteins, transporters, and sensor/regulators in continuous, multi-gene clusters known as Polysaccharide Utilization Loci (PULs (37)). The complement of PULs in a bacterium dictates its carbohydrate utilization profile and thus contributes to fitness in a given environment. PULs have been predicted bioinformatically in a number of human gut *Segatella* and, in some cases, broadly correlated with growth on individual plant polysaccharides (38–43). However, many predicted *Segatella* PULs lack experimentally defined genomic boundaries or are otherwise ambiguous regarding their full composition, which hinders cross-genome analysis and prediction of metabolic function. Furthermore, it is presently unclear to what extent the respective PUL repertoires of specific *Segatella* versus *Bacteroides* species might cause direct competition or enable cooperation, leading to selection in individual HGM mediated by diet.

Hence, in the present study, we used RNA sequencing (RNA-seq) to delineate a reference set of plant polysaccharide-specific PULs in the type-strain *Sc*DSM18205, analogous to seminal work on human gut *Bacteroides* (44). Building upon this foundation, we examined the temporal transcriptional response of *Sc*DSM18205 following exposure to individual polysaccharides and a mixture of plant cell wall polysaccharides to explore the extent of substrate prioritization for growth (45, 46). Finally, we co-cultured *Sc*DSM18205 with *Bacteroides thetaiotaomicron* or *Bacteroides ovatus* to reveal how individual PUL complements dictated coexistence or competition on specific polysaccharides.

## RESULTS

### Transcriptomic definition of plant polysaccharide utilization systems

Putative polysaccharide utilization loci (PULs) in *Sc*DSM18205 have been predicted computationally, including current entries in the Polysaccharide-Utilization Loci DataBase (PULDB) (39–41, 43). To experimentally validate *Sc*DSM18205 PULs responsible for the metabolism of the cell wall and storage polysaccharides of fruits, vegetables, and grains, we performed comparative growth studies and RNA-seq. Concordant with previous studies (41, 43), robust growth was observed in modified peptone-yeast media (mPYM) using several plant polysaccharides as sole carbohydrate sources (Fig. S1). These included β-glucans (e.g.*,* barley mixed-linkage β(1, 3)/β(1, 4)-glucan, tamarind xyloglucan), xylans (beechwood glucuronoxylan, wheat arabinoxylan, corn xylan oligosaccharides), pectic polysaccharides (citrus homogalacuronan, potato rhamnogalacturonan I, potato galactan, sugar beet arabinan), and chicory β(1, 2)-fructan (inulin). No growth was observed on β-mannans (konjac glucomannan, carob galactomannan), β(1, 3)-galactans (gum Arabic, larch arabinogalactan), microbial β(1, 3)-glucans (*Alcaligenes faecalis* curdlan, *Laminaria digitata* laminarin, *Lentinula edodes* lentinan), and α-glucans (potato amylose, maize amylopectin, *Leuconostoc* spp. dextran, *Aureobasidium pullulans* pullulan, bovine liver glycogen) (data not shown).

**Fig 1:**
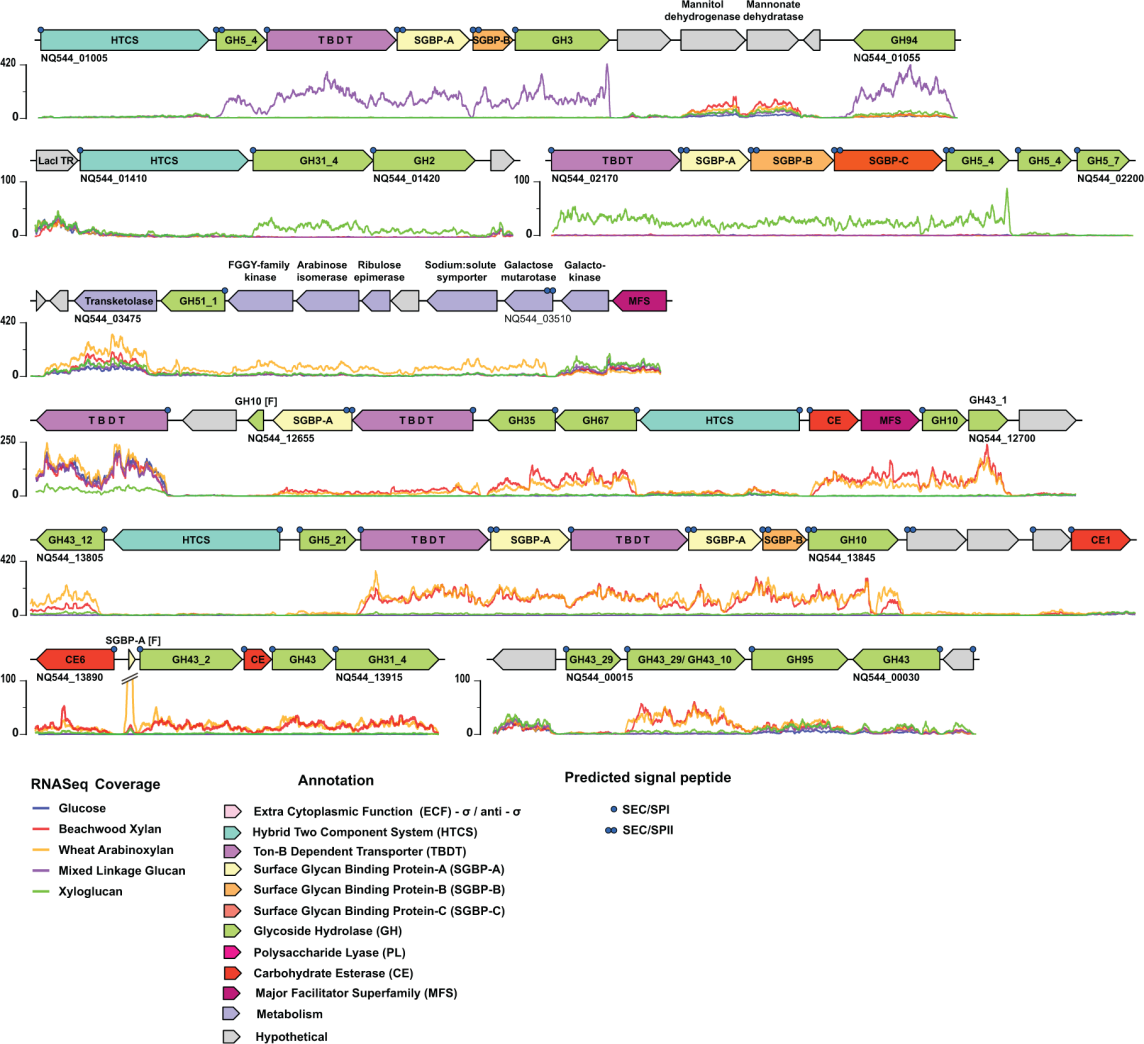
Hemicellulose-associated polysaccharide utilization loci and CAZyme clusters in *Sc*DSM18205. Per-nucleotide base coverage by RNAseq for cultures grown on the indicated carbohydrates is indicated below each gene locus. Genes are colored according to the key, based on predicted protein annotations. Predicted signal peptides are denoted on individual genes as follows: •, signal peptidase I cleavage; ••, signal peptidase II cleavage (N-terminal Cys, lipidated).

**Fig 2:**
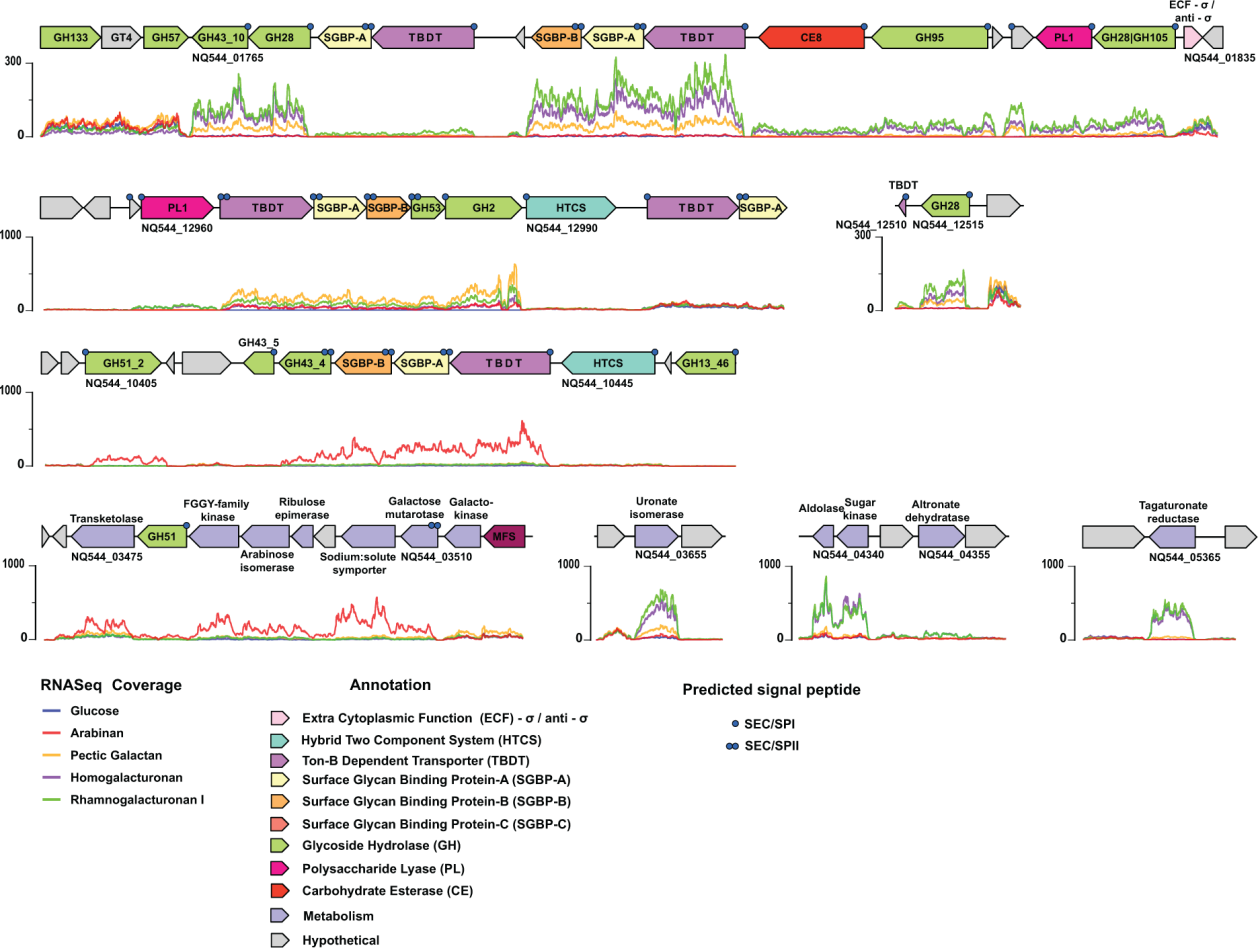
Pectin-associated polysaccharide utilization loci and CAZyme clusters in *Sc*DSM18205. Per-nucleotide base coverage by RNA-seq for cultures grown on the indicated carbohydrates is indicated below each gene locus. Genes are colored according to the key, based on predicted protein annotations. Predicted signal peptides are denoted on individual genes as follows: •, signal peptidase I cleavage; ••, signal peptidase II cleavage (N-terminal Cys, lipidated).

**Fig 3:**
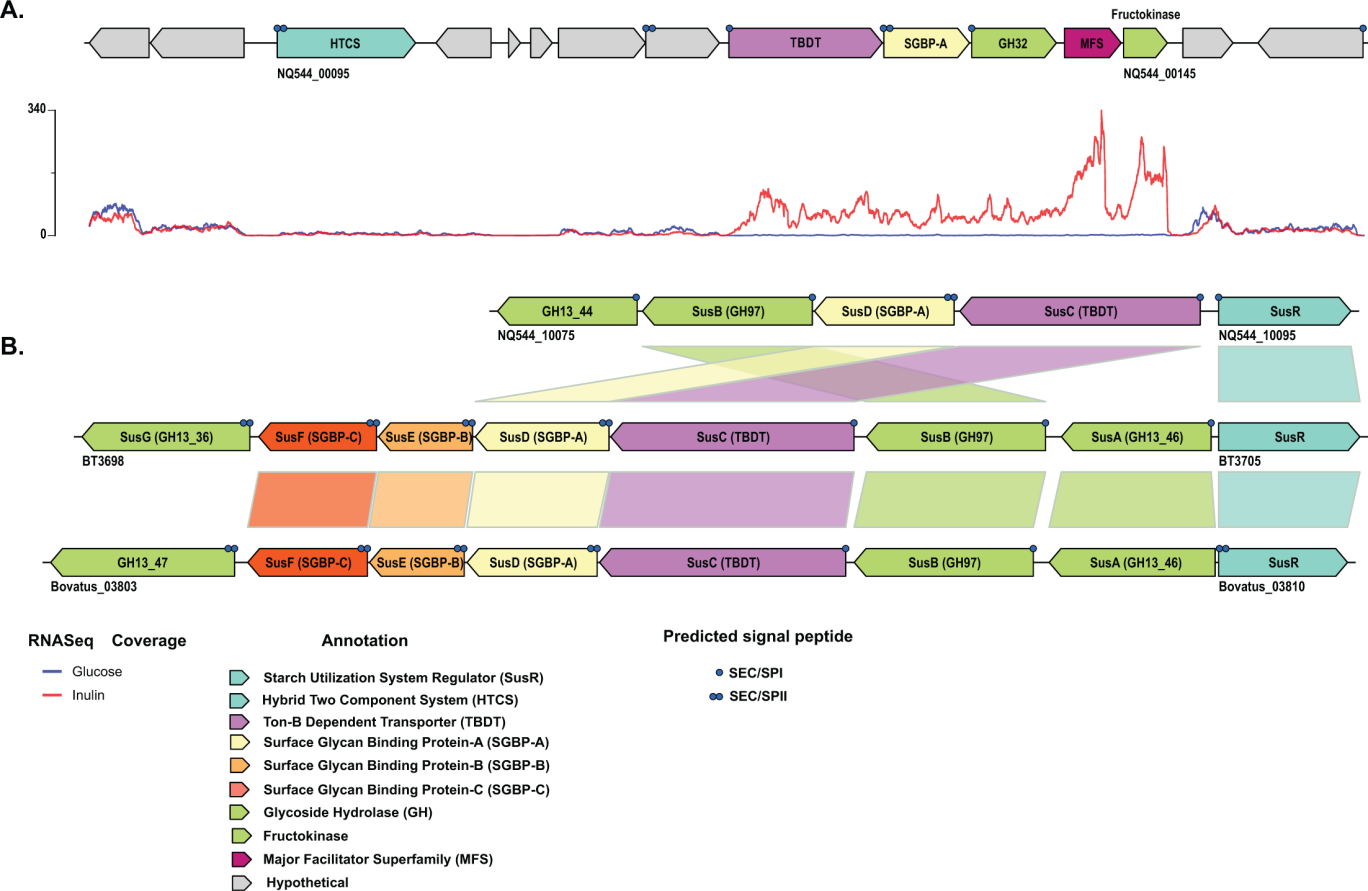
Storage polysaccharide-associated polysaccharide utilization loci in *Sc*DSM18205. (**A**) Inulin PUL. Per-nucleotide base coverage by RNA-seq for cultures grown on the indicated carbohydrates is indicated below each gene locus. Genes are colored according to the key, based on predicted protein annotations. (**B**) Predicted starch utilization system (Sus) genes in *S. copri* DSM18205 versus *B. thetaiotaomicron sus* homologs. Predicted signal peptides are denoted on individual genes as follows: •, signal peptidase I cleavage; ••, signal peptidase II cleavage (N-terminal Cys, lipidated).

Differential RNA-seq analysis of exponential-phase cultures grown in mPYM enabled us to define the boundaries of cognate PULs, and in some cases, associated “CAZyme Clusters” (40), for mixed-linkage β-glucan, xyloglucan, xylans, pectic polysaccharides, and inulin (Fig. 1 to 3; Fig. S2). Notably, the polysaccharide utilization systems encoded by *Sc*DSM18205 exhibit significant differences from their functionally characterized counterparts in *Bacteroides* species (16, 47, 48).

#### Mixed-linkage β-glucan utilization

Mixed-linkage β(1, 3)/β(1, 4)-glucans are abundant in cereals such as barley, rye, and oats, in which they can constitute up to 70% by weight of the grain endosperm, thereby comprising a key component in staple human diets (49). Notably, consumption of barley kernel-based food has been shown to enrich *Prevotellaceae* in mouse and human gut microbiota (29). During growth of *Sc*DSM18205 on barley mixed-linkage β-glucan, a contiguous group of genes (NQ544_01010–01030) was upregulated (Fig. 1; Fig. S2A). Including an associated, non-upregulated hybrid two-component system (HTCS)-encoding gene (NQ544_01005), these correspond to predicted PUL4 in the PULDB (40). The encoded proteins include a predicted starch utilization system SusC/SusD-homolog pair (SusC_H_/SusD_H_, i.e., a Ton-B-dependent transporter [TBDT] and a surface glycan binding protein [SGBP-A]), an accessory SGBP-B, a glycoside hydrolase family 5 (GH5) subfamily four member (GH5_4, putative cell-surface *endo*-glucanase), and a GH3 member (putative periplasmic β-glucosidase).

This PUL is structurally similar to one from *B. ovatus* (Bovatus_03148–03153 (40)), which was shown to facilitate the complete degradation and utilization of mixed-linkage β-glucan (50). The primary difference between the *B. ovatus* and *Sc*DSM18205 mixed-linkage β-glucan PULs is that *B. ovatus* encodes a GH16 *endo*-β(1, 3)/β(1, 4)-glucanase to initiate polysaccharide backbone hydrolysis (50), whereas *S. copri* encodes a GH5_4 member (51), which we have likewise shown to be *endo*-β(1, 3)/β(1, 4)-glucan-specific (52). We have also recently experimentally demonstrated that the GH3 member is a β-glucan oligosaccharide hydrolase (β-glucosidase) (52). A gene (NQ544_01055) encoding a GH94 member (predicted disaccharide phosphorylase) was not considered to be part of the *Sc*DSM18205 mixed-linkage β-glucan-PUL because of its distal location and opposite orientation in the chromosome (Fig. 1).

#### Xyloglucan utilization

The xyloglucans are a family of highly branched polysaccharides that are abundant in the cell walls of vascularized plants (53), especially including dicot vegetables and fruits in the human diet. Xyloglucans have a β(1, 4)-glucan backbone that is heavily branched with α(1, 6)-xylosyl residues, which can be further extended with a range of additional monosaccharides, dependent on species and tissue of origin (54). *Bacteroides* xyloglucan utilization systems are encoded by single, contiguous PULs (Bovatus_03064–03075 (40)), whose diversity of GHs reflects this structural complexity (55, 56). In contrast, the xyloglucan utilization system in *Sc*DSM18205 appears to be encoded in a core PUL (NQ544_02170–02195) and an accessory CAZyme Cluster (NQ544_01410–01420; Fig. 1; Fig. S2B).

The core xyloglucan PUL encodes a TBDT/SGBP complement, notably including two predicted accessory SGBPs (-B and -C), together with two GH5_4 members (predicted *endo*-xyloglucanases), the latter of which is weakly upregulated. This group corresponds to predicted PUL7 in the PULDB (40); however, we exclude here the GH5_7 member because of a lack of upregulation (Fig. 1) and the observation that all members of GH5_7 characterized to date are mannanases (57).

The co-regulated CAZyme Cluster (NQ544_01410–01420, not identified in the PULDB) encodes an HTCS regulator (not upregulated), a GH2 member (predicted β-galactosidase), and a GH31 member (predicted α-xylosidase), consistent with core xyloglucan sidechain hydrolysis. The identities and specific contribution of homologous fucosidases and arabinofuranosidases in *Sc*DSM18205 required for terminal sidechain hydrolysis (55, 56) is still unclear, however. For example, the most upregulated GH95 (potential α-fucosidase or α-L-galactosidase)-encoding gene is NQ544_00025 (Fig. 1); however, this is only upregulated ca. 3-fold on xyloglucan versus glucose (Fig. S2B). Similarly, most GH43 (predicted α-arabinofuranosidase)-encoding genes are not differentially regulated on xyloglucan (Fig. 1). Although some questions remain, these data together provide broader insight into the composition of the xyloglucan utilization system in *Sc*DSM18205 and related strains than obtained by previous genome and *tbdt*-specific transcript analysis (41).

#### Xylan utilization

Xylans are represented in the human diet predominantly as (glucurono)arabinoxylans found in cereal grains and, to a limited extent, dicot cell walls (53, 58). The transcriptional response of *Sc*DSM18205 on wheat arabinoxylan and beechwood glucuronoxylan (a non-arabinosylated, secondary cell wall xylan) was essentially identical (Fig. 1; Fig. S2C and D). The data revealed the upregulation of a complex xylan utilization system comprising two *susC/susD*-homolog-containing PULs and two CAZyme Clusters, which encode a diverse complement of xylan backbone and sidechain-cleaving GHs with characterized homologs in *Bacteroides* species (59–62). However, the architecture of these PULs and clusters was distinct, lacking synteny to previously characterized PULs from *Bacteroides* (62).

The most strongly upregulated PUL (NQ_13805–13865) corresponds precisely to predicted PUL17 in the PULDB (40) and notably encodes a tandem pair of SusC/SusD-homologs, a predicted GH10 *endo*-xylanase, and a recently characterized α-L-arabinofuranosidase active on mono-substituted backbone xylosyl residues (63). Constitutively expressed genes encoding an HTCS and a GH5_21 predicted *endo*-xylanase are also part of this PUL. This pattern of regulation directly recapitulates that of an exactly syntentic xylan PUL in the rumen isolate *Prevotella bryantii* B_1_4 (64). Syntenic PULs from *S. copri* HDD04 and a human fecal metagenome assembled genome have been correlated with growth on arabinoxylan (43) and fitness on vegetable-rich diets (42), respectively.

The second PUL (NQ_12660–12700) encompasses most of predicted PUL15 in the PULDB (40) but was shown here to terminate after a weakly upregulated *susC/susD* homolog pair and before a truncated GH10-encoding gene (NQ544_12655, Fig. 1). These observations may suggest that this is a partially redundant PUL, which nonetheless supplies key xylan debranching enzymes, including a predicted carbohydrate esterase. For example, the GH10 and GH43_1 members were recently biochemically validated to be an *endo*-β-xylanase and an *exo*-β-xylosidase, respectively (63). This PUL also includes a major facilitator superfamily (MFS) protein (NQ544_12690), which, in this context, may transport monosaccharides from the periplasm to the cytoplasm (65). Notably, each xylan PUL encodes a HTCS regulator (44, 66), whose specificities are currently unknown. In the corresponding *B. ovatus* system (Bovatus_03715–03744 and Bovatus_01723–01732 (40)), the two xylan PULs were upregulated differently in response to arabinoxylan versus glucuronoxylan (62); however, this was not the case with *Sc*DSM18205 (Fig. 1).

The two putative CAZyme clusters were weakly upregulated on the basis of per-nucleotide counts (Fig. 1), with some genes (e.g., NQ544_00015, GH43) lacking significant expression (*P* > 0.05, Fig. S2C and D). Nonetheless, these clusters encode several GH43 members (Fig. 1), including the moderately upregulated NQ544_00020 and a GH95 member (potential α-L-galactosidase), implicating them in xylo-oligosaccharide deconstruction (62). We also observed that a cluster of genes (NQ544_033475–03510) encoding an arabinose metabolic pathway was specifically upregulated on arabinoxylan, but not glucuronoxylan (Fig. 1). Notably, this cluster (minus the GH51-encoding gene) was also upregulated on pectic arabinan, but not other pectic polysaccharides (Fig. 1, see below).

#### Pectin utilization

Pectin is a complex plant cell wall polysaccharide that is rich in galacturonic acid (GalA). Pectin is widespread in the human diet from dicot plants, especially fruits, and to a lesser extent, in cereals (67). The simplest pectic polysaccharide is homogalacturonan, which consists of up to 100 α(1, 4)-linked GalA residues. Rhamnogalacturonan I consists of alternating α(1, 4)-GalA and α(1, 2)-rhamnosyl residues and is believed to be contiguous with homogalacturonan domains. In the plant cell wall, rhamnogalacturonan I is typically branched by complex arabinan, galactan, or arabinogalactan side chains at O-4 of half of the rhamnosyl residues (68). Rhamnogalacturonan II results from uniquely complex branching of homogalacturonan with four sidechains comprising 12 different glycosyl residues (67–69). The structural complexity and covalent links of pectic polysaccharides make their isolation in pure form difficult.

Corresponding to this compositional diversity, a large number of CAZyme-, SGBP-, and TBDT-encoding genes across several genomic regions were upregulated in response to homogalacturonan, rhamnogalacturonan I, pectic galactan, and/or pectic arabinan (Fig. 2; Fig. S2E through H). The CAZyme complement, which included diverse GH, polysaccharide lyase (PL), and carbohydrate esterase (CE) families, had predicted activities consistent with pectin degradation, informed in part by previous enzymology of *Bacteroides* homologs (70) (arabinan, BT0348-0369; homogalacturonan, BT4108-4124; galactan, BT4667-4673; rhamnogalacturonan I, BT4145-4183 (40)). Notably, however, the pectin utilization system of *Sc*DSM18205 comprised many fewer components than in *B. thetaiotaomicron* (Fig. 2; cf. Fig. 1 in reference 70), despite growth on the same pectic polysaccharides. There was also a lack of synteny between both systems, with the exception of a PUL implicated in pectic galactan saccharification (NQ544_12965–12985, part of Predicted PUL16 (40)) and a cluster of arabinose metabolism genes specifically upregulated on pectic arabinan (NQ544_03475–03510, Fig. 2). These differences are notable in light of the *Bacteroidacae*/*Prevotellacae* evolutionary split.

Our transcriptomic data also allow the refinement of PUL boundaries from those originally predicted bioinformatically (38, 40). Growth on homogalacturonan and rhamnogalacturonan I revealed that predicted PUL5 in fact terminates at NQ544_01765 and excludes subsequent GH133, glycosyl transferase family 4 (GT4), and GH57 (predicted α-glucan-related enzymes). High upregulation of NQ544_01770 (GH28, predicted (rhamno)galacturonidase) and NQ544_01765 (GH43_10, predicted α-L-arabinofuranosidase) is concordant with core pectin utilization (Fig. 2). Unusually, the upstream *susC/susD* (*tbdt/sgbp*) homolog pair (NQ544_01780, NQ544_01775) is only weakly upregulated. However, high upregulation is observed for the TBDT/SGBP-A/SGBP-B trifecta encoded just upstream in predicted PUL6 (NQ544_01790–01810), which also includes upstream genes encoding CE8 (predicted pectin methylesterase) and GH95 (predicted α-L-galactosidase) members. These *susC/susD* homolog pairs are separated by an apparent gene fragment, rationalizing why automated annotation placed them in separate PULs. Further upstream, genes encoding a PL1 member (predicted pectate lyase, NQ544_01825) and tandem GH28-GH105 members (predicted (rhamno)galacturonidase-4,5-unsaturated hexenuronidase, NQ544_01830) were also upregulated on homogalacturonan and rhamnogalacturonan I. Together, these data suggest that these genes function as one large PUL dedicated to pectin backbone breakdown. A lone gene encoding a GH28 member (NQ544_12515, predicted *endo*-galacturonidase) was also upregulated and implicated in pectin backbone cleavage (Fig. 2)

Correspondingly, several genes involved in uronic acid metabolism were particularly highly upregulated during growth on homogalacturonan and rhamnogalacturonan I. These included genes encoding a uronate isomerase (NQ544_03655), a tagaturonate reductase (NQ544_05365), a bifunctional 4-hydroxy-2-oxoglutarate aldolase/2-dehydro-3-deoxy-phosphogluconate aldolase (NQ544_04340), and a sugar kinase (NQ544_04345). Nearby genes (NQ544_04350–04360) encoding a LacI-like binding protein, altronate dehydratase, and antiporter were not upregulated on pectins (Fig. 2).

We were also able to delineate PULs mediating pectin sidechain, *viz*. arabinan and galactan, degradation. Growth of *Sc*DSM18205 on pectic galactan resulted in the specific upregulation of a PUL (NQ544_12965–12985) encoding a TBDT (SusC homolog), SGBP-A (SusD homolog), SGBP-B, a GH53 member (predicted *endo*-galactanase), and a GH2 member (predicted exo-β-galactosidase). Neither a downstream HTCS-encoding gene (NQ544_12990) nor an upstream PL1-encoding gene (NQ544_12960) was regulated (Fig. 2; Fig. S2G). Notably, this latter gene was upregulated during growth on homogalacturonan and RG-I, consistent with a role in the specific cleavage at galacturonate groups in the pectin backbone. The aforementioned genes are all contained within predicted PUL16, which also includes another *susC/susD* homolog-containing region (NQ544_12995–13040 and NQ544_12960). Taken together, our present transcriptomic data allow us to redefine the galactan-PUL of *Sc*DSM18205 as comprising only the loci NQ544_12965–12985 and possibly NQ544_12990.

Growth on pectic arabinan resulted in high upregulation of NQ544_10425–10440, encoding a predicted GH43_4 member (*endo*-arabinanase), an SGBP-B, an SGBP-A (SusD homolog), and a TBDT (SusC homolog), as well as NQ544_10405, encoding a predicted GH51_2 member (α-L-arabinofuranosidase). Interestingly, an intervening GH43_5-encoding gene (predicted *exo*-α−1,5-L-arabinofuranosidase) was not upregulated (Fig. 2; Fig. S2H) but is otherwise found in several *S. copri* isolates (43). These genes comprise part of Predicted PUL11 (40), which also contains several α-glucan-related genes that are clearly not relevant to pectin utilization (e.g., NQ544_10455–10470), as supported by the present RNA-seq data. As noted above, growth on pectic arabinan also resulted in the specific upregulation of a cluster of arabinose metabolic genes (NQ544_03475–03510, except NQ544_03480; Fig. 2 cf. Fig. 1).

#### Inulin utilization

Inulin is a homopolymer of β(1, 2)-linked fructosyl units, which functions as storage polysaccharide in many vegetables and is ubiquitous and abundant in human diets (71). Inulin-induced upregulation of a cluster of genes (NQ544_00095–00145) that not only encompassed predicted PUL1 in the PULDB (40), but also included genes encoding a MFS transporter and a fructokinase (Fig. 3A; Fig. S2I). This minimalist PUL included a canonical *susC/susD*-homolog pair and a single CAZyme from GH32 (predicted *endo*-inulinase), but was smaller (regarding GH32 and SGBP members) and had different organization than related inulin and levan PULs from *Bacteroides* species (BT1754-1765 and Bovatus_01822–01832) (72–74) and *S. copri* HDD04 (43). A contiguous HTCS-encoding gene was not observed, and the association of a homolog upstream of the inulin PUL (NQ544_00085, Fig. 3A) is currently unclear.

#### Lack of starch utilization

Starch, comprising the linear α(1, 4)-glucan amylose and α(1, 6)-branched amylopectin, is arguably the most important plant storage polysaccharide and major energy source in human diets globally. Although hydrated starch is generally readily converted to glucose by human amylases and α-glucosidases, morphologically recalcitrant forms known as “resistant starches” are known to reach the lower gastrointestinal tract (75), where they are metabolized by diverse members of the HGM (76). Indeed, the archetype of the Bacteroidetes PUL paradigm is the starch utilization system of *B. thetaiotaomicron,* encoded by encoded by *susABCDEFG*/*R* (77).

In light of the ubiquity of the Sus among Bacteroidetes (34, 40), our inability to observe growth of *Sc*DSM18205 on starch was indeed surprising but might be explained by the apparent degeneracy of the homologous PUL in this strain. As shown in Fig. 3B, analysis of synteny between the *B. thetaiotaomicron sus* genes and *Sc*DSM18205 reveals a lack of a homologous cell-surface *endo*-amylase in the latter, which would be critical for initiating extracellular polysaccharide cleavage. In contrast, *Sc*DSM18205 exhibited robust growth on maltose, competent growth on maltotriose, and weak growth on maltopentaose and maltohexaose in our hands (Fig. S4). Hence, we posit that a previous report indicating limited starch utilization by *Sc*DSM18205 may have been a consequence of long incubation times (up to 72 h [41]), resulting in the eventual release of periplasmic α-glucosidases, of which there are several encoded by the *Sc*DSM18205 genome (GH13_44, GH13_14, GH13_46, 2 × GH97, cf. references [77, 78]), into the culture medium.

### Short-chain fatty acid production

The *Sc*DSM18205 genome encodes all the enzymes for the Embden–Meyerhoff–Parnas pathway to produce pyruvate for SCFA anabolism; however, unlike *Bacteroides* species, it lacks three key enzymes required to generate propionate from succinate (79). Previous studies have indeed shown that *Sc*DSM18205 primarily produces succinate and acetate, together with a limited amount of formate, when grown on glucose (a hexose) and xylose (a pentose). Moreover, it was also shown that growth on xylose resulted in increased succinate and acetate production (80). Therefore, we grew *Sc*DSM18205 on individual polysaccharides as sole carbohydrate sources and quantified SCFA levels to assess whether complex polysaccharide composition influences the metabolic output of the bacterium (Fig. S3).

As expected, succinate and acetate were the major SCFA products along with minor amounts of butyrate. Neither propionate nor valerate was observed. Concentrations of succinate and acetate generally increased from the early exponential growth phase through the mid-exponential and stationary phases and declined slightly in the late-stationary phase (Figure S3A through D). With all polysaccharides, succinate showed the greatest increase throughout the exponential and early stationary phases, with only a slight decrease in the late stationary phase; acetate levels were more varied, peaking in the stationary phase and then falling in the late-stationary phase. Similarly, *Bacteroides* species also showed reduced acetate levels in the late stationary phase (81).

No conclusive differences in succinate levels were observed among different polysaccharides, although inulin, pectic arabinan, and pectic galactan produced marginally higher amounts in the stationary phase. On the other hand, some pectic polysaccharides (e.g., homogalacturonan and rhamnogalacturonan I) resulted in higher, yet variable, acetate production throughout growth. These differences may result from the metabolism of galacturonic acid released from their backbones, which utilizes an alternative pathway in Gram-negative bacteria (82). We note that acetate plays a pivotal role in generating ATP in muscle tissue and lipogenesis, whereas succinate is central to intestinal gluconeogenesis and contributes toward improved body weight (83, 84). Also, a higher level of succinate was found to be associated with vegetarian diets (85), which are known to enrich the HGM with *Prevotellaceae* (11, 21, 27, 86).

### Temporal transcriptional profiling of polysaccharide utilization

Previous studies of *Bacteroides* species (family *Bacteroidaceae*) have illuminated how specific transcriptional responses mediate glycan prioritization in complex mixtures and enable co-existence in mixed populations (45, 46, 87). Inspired by this seminal work, we sought to reveal to what extent *Sc*DSM18205, as an exemplar *Prevotellaceae*, might similarly prioritize the metabolism of individual polysaccharides in a time-dependent manner. Ultimately, we were interested in understanding how polysaccharide prioritization might affect competition between *Sc*DSM18205 and *Bacteroides* species in light of the distinction of *Prevotellaceae*- and *Bacteroidaceae*-rich “enterotypes” in individual HGM.

#### Medium optimization

Consistent with previous studies (34, 41, 43), we observed that cultivation of *Sc*DSM18205 in mPYM (used for our RNA-seq experiments, see above) commonly resulted in extended lag phases with polysaccharides (Fig. S1). To expedite subsequent temporal transcriptional profiling and growth competition experiments (see below), we optimized the yeast casitone fatty acid (YCFA) basal medium by increasing vitamin levels and removing yeast extract. The modified medium (mYCFA, Table S1) resulted in a reduced lag phase, faster growth rate, and higher cell density versus mPYM (Fig. S4; Table S3). The overall profile of polysaccharide utilization by *Sc*DSM18205 in mYCFA was unchanged versus mPYM (Fig. S4 cf*.* Fig. S1). Importantly, for subsequent co-culture experiments (see below), mYCFA was also compatible with the growth of *B. thetaiotaomicron* and *B. ovatus* (Table S3).

#### Transcriptional response on individual plant β-glucans

**Fig 4:**
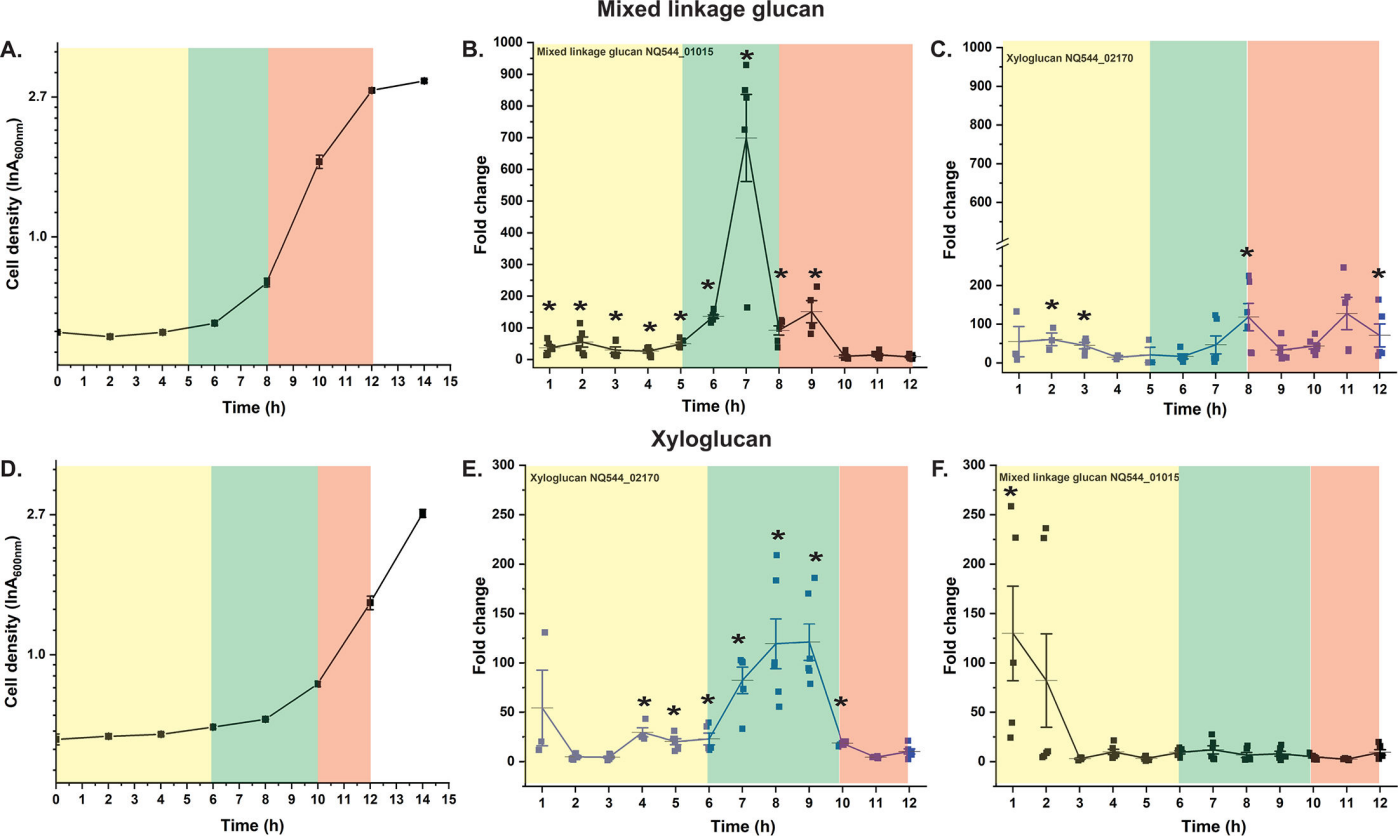
Growth kinetics and temporal expression of *Sc*DSM18205 *tbdt* genes in mixed-linkage β-glucan and xyloglucan as sole polysaccharide sources. (**A**) Growth on mixed-linkage β-glucan (2.5 g/L). (**B and C**) Transcript levels of cognate mixed-linkage β-glucan (NQ544_01015) and xyloglucan (NQ544_02170) *tbdt* genes in response to mixed-linkage β-glucan. (**D**) Growth on xyloglucan (2.5 g/L). (**E and F**) Transcript levels of cognate xyloglucan (NQ544_02170) and mixed-linkage β-glucan (NQ544_01015) *tbdt* genes in response to xyloglucan. All transcript level changes are relative to levels prior to exposure to the polysaccharide ( = 0). Error bars represent the SEM of three technical replicates from a single biological sample. Asterisks indicate data points with upregulation significantly greater than a basal level (set at 10-fold), as determined by an unpaired *t* test. Background colors delineate lag (yellow), early exponential (green), and exponential (salmon) growth phases. For extended-duration growth profiles, see Figure S4.

Analogous to previous studies, we used RT-qPCR to monitor transcript levels of *tbdt* (*susC* homolog) genes of individual PULs as indicators of expression during growth (44–46, 87). In preliminary experiments, we noted that extraction of RNA from *Sc*DSM18205 was more challenging and led to lower yields than with *Bacteroides* species, particularly in the lag phase, necessitating the use of higher cell densities (OD_600_ 0.5) in initial innocula (Fig. 4). Using the representative β-glucans from monocots and dicots, mixed-linkage β-glucan and xyloglucan, respectively, we measured the transcript levels of *tbdt* genes associated with the utilization of mixed-linkage β-glucan, xyloglucan, pectic polysaccharides, xylan, inulin, and starch throughout the lag and exponential phases of growth. Data were normalized to transcript levels of cells grown in glucose medium after washing with a carbohydrate-free medium, prior to transfer to mixed-linkage β-glucan or xyloglucan medium. On both polysaccharides, we observed a rapid, initial upregulation of select *tbdt* genes associated with mixed-linkage β-glucan, xyloglucan, xylan, pectin, and inulin utilization in the absence of growth (Fig. 4; Fig. S5). In control experiments, in which washed *Sc*DSM18205 cells were transferred back into the medium with glucose as a sole carbohydrate source, upregulation of these *tbdt* genes was not observed (Fig. S6). The levels of initial upregulation on mixed-linkage β-glucan and xyloglucan were variable, and in many cases, transient; however, the non-cognate *tbdt* genes exhibited some level of constitutive expression through the growth phases. A notable exception was the *tbdt* from the degenerate starch PUL (NQ544_10090, Fig. 3B), which was unresponsive (Fig. S5F). Together, these data suggest that several PULs in *Sc*DSM18205 are subject to classic catabolite repression by glucose, which is relieved rapidly upon transfer to polysaccharide media.

Regarding the response of the mixed-linkage β-glucan and xyloglucan PULs on their cognate polysaccharides, initial transient periods of upregulation were followed by relaxation in the lag phase, similar to other PULs (Fig. 4). Distinctly, *tbdt* transcription spiked sharply at the transition between the lag and exponential phases (5–8 h on mixed-linkage β-glucan, 6–10 h on xyloglucan) on the respective substrates before again relaxing back to basal levels. These results were intriguing and suggested that regulation is driven by early-stage sensing of polysaccharide hydrolysis products, which are produced and imported by constitutive levels of PUL protein machinery (88). We predict that the corresponding HTCS sensor/regulators are encoded by NQ554_01005 (mixed-linkage β-glucan) and NQ544_01410 (xyloglucan), based on their proximity to genes encoding other utilization system components (Fig. 1). Remarkably, PUL protein synthesis, as reflected by transcription, appears to occur intensively only during a brief window at the late-lag/early-exponential phase transition. After this, transcript levels drop sharply in the exponential phase, that is, after 7 h for the mixed-linkage β-glucan PUL during growth on mixed-linkage β-glucan and after 9 h for the xyloglucan PUL during growth on xyloglucan. This suggests that daughter cells maintain sufficient levels of PUL proteins to sustain growth, without requiring the production of additional copies of the PUL machinery after division. The shutting down of mixed-linkage β-glucan and xyloglucan PUL expression early in exponential-phase growth is also consistent with catabolite repression by glucose, a saccharification product of both polysaccharides.

Analysis of polysaccharide hydrolysis products in the medium by HPLC and TLC during growth on mixed-linkage β-glucan or xyloglucan was concordant with these observations (Fig. S7). No oligosaccharides were observed in the lag phases, prior to PUL upregulation. Interestingly, at 7 h, when PUL expression was high but growth was still minimal, detectable levels of oligosaccharides were observed in the media (Fig. S7) along with much polysaccharide (Fig. S7B and C, TLC baseline). At 9 h for mixed-linkage β-glucan and 10 h for xyloglucan (corresponding to early exponential-phase growth and minimal PUL expression; Fig. 4), the polysaccharides had been fully hydrolyzed to oligosaccharides. These were mostly consumed by 12–14 h. In the case of mixed-linkage β-glucan, only a trace amount of cellobiose remained at the end of exponential phase growth (13 h, Fig. S7B) likely because cellobiose is only slowly utilized by *Sc*DSM18205, Fig. S4).

#### Transcriptional response to a complex polysaccharide mixture

The human diet is diverse and varied among populations and individuals, yet typically includes a mixture of plant cell wall polysaccharides from fruits, vegetables, and grains. Building upon the initial understanding of transcriptional regulation on single β-glucans, we were particularly interested in exploring the response of *Sc*DSM18205 to an ensemble of plant polysaccharides. Hence, we grew *Sc*DSM18205 on a mixture comprising equal amounts (wt/vol) of barley mixed-linkage β-glucan, tamarind xyloglucan, corn xylan oligosaccharides, citrus homogalacturonan, potato rhamnogalacturonan I, sugar beet arabinan, chicory inulin, and soluble starch. Initial screening in microtiter plates using total polysaccharide concentrations of 0.1, 0.5, 1, 2, and 8 g/L (i.e., individual polysaccharide concentrations were one-eighth these values) revealed carbohydrate-limited growth at all concentrations except 8.0 g/L, versus growth on 5 g/L glucose (Fig. S8). Similar to growth on glucose, essentially no lag phase was observed with the polysaccharide mixture, which we attribute to rapid growth on inulin and corn xylan oligosaccharides (Fig. S8 cf. Fig. S4).

**Fig 5:**
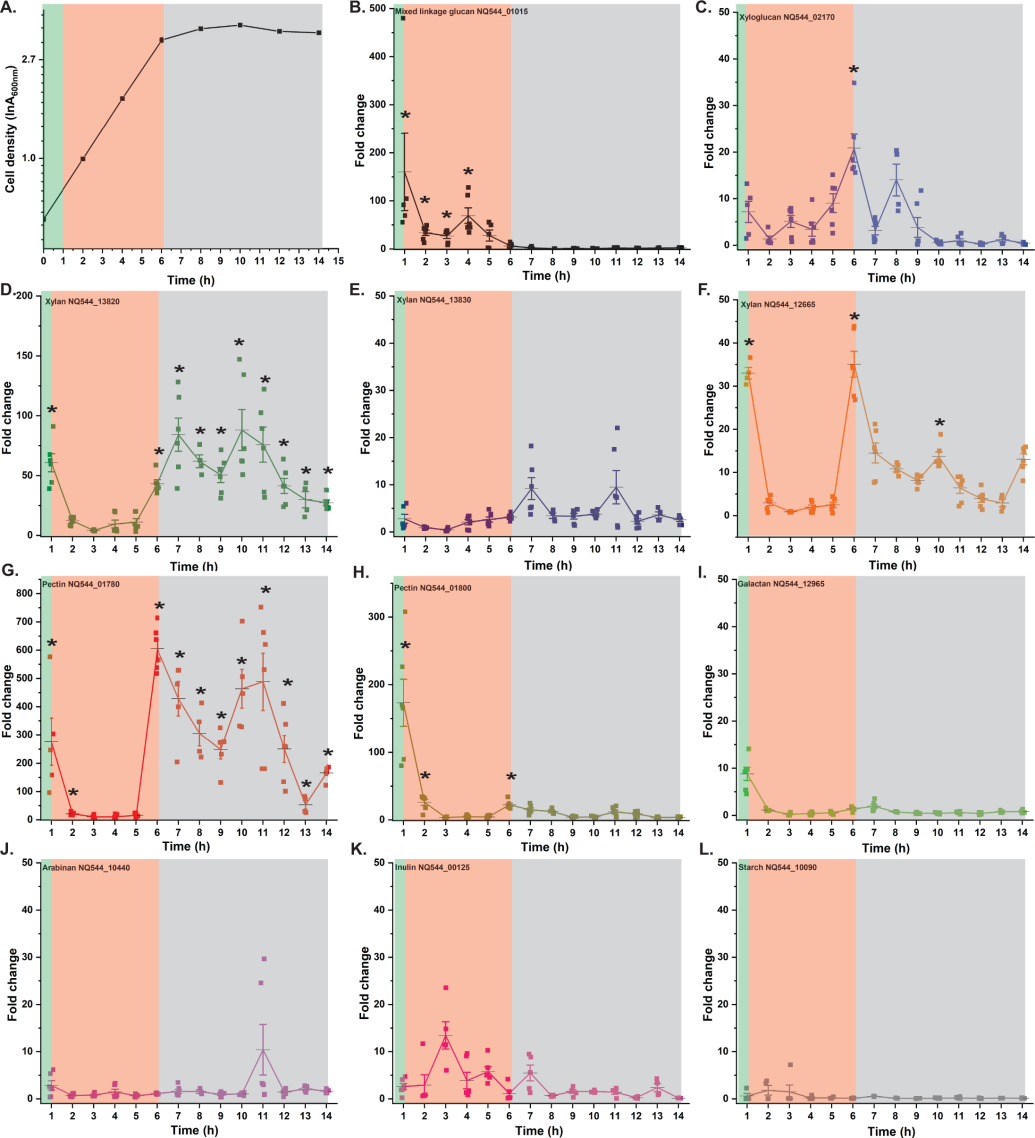
Growth kinetics and temporal expression of *Sc*DSM18205 *tbdt* genes in mYCFA supplemented with a mixture of dietary plant polysaccharides. The medium contains equal concentrations of barley mixed-linkage β-glucan, tamarind xyloglucan, corn xylan oligosaccharides, citrus homogalacturonan, potato rhamnogalacturonan I, sugar beet arabinan, chicory inulin, and soluble starch (0.625 g/L each, total concentration 5 g/L). (**A**) Growth kinetics. Individual data are means of two biological replicates. (**B-L**) Temporal expression of individual polysaccharide-associated *tbdt* genes. All transcript level changes are relative to time zero, that is, prior to transfer to the mixture. Error bars represent the SEM of three technical replicates from a single biological sample. Asterisks indicate data points with upregulation significantly greater than a basal level (set at 10-fold), as determined by an unpaired *t* test. Background colors delineate early exponential (green), exponential (salmon), and stationary (grey) growth phases.

Based on these data, we scaled-up the growth using 5 g/L of the polysaccharide mixture to enable monitoring of *tbdt* transcript levels by RT-qPCR over 14 h, comprising the entire log and stationary phases (Fig. 5A). The resulting data (Fig. 5B through I) revealed that specific pectin-backbone- (NQ544_01780 and NQ544_01800), xylan- (NQ544_13820), and mixed-linkage β-glucan-associated (NQ544_01015) *tbdt* genes were rapidly and highly upregulated (50-fold to 300-fold) at the onset of exponential-phase growth (*t* = 1 h), followed by a return to baseline levels during the mid- to late-exponential phases. Notably, the pectin-backbone- and xylan-associated *tbdt* genes, NQ544_01780 and NQ544_13820, respectively, were again highly upregulated during the stationary phase. Other specific xylan- (NQ544_12665) and xyloglucan-associated (NQ544_02170) *tbdt* genes exhibited a similar two-peak pattern of upregulation, albeit with lower fold-change maxima (10-fold to 40-fold). The galactan-associated *tbdt* (NQ544_12965) was weakly co-upregulated with the pectin-backbone-associated *tbdt* genes in the early-exponential phase but returned to baseline levels for the duration of the experiment (*n.b*., pectic galactan was not explicitly added in the mixture but was present as in impurity in other pectic polysaccharides). Other *tbdt* genes did not appear to be upregulated during this extended experiment, including those associated with xylan (NQ544_13830) and pectic arabinan (NQ544_10440). Likewise, the *tbdt* (NQ544_10090) from the degenerate Sus (Fig. 3) was not upregulated and served as a clear negative control. Distinctly, the inulin-associated *tbdt* (NQ544_00125) was primarily, but weakly (10-fold), upregulated during the mid-exponential phase in this experiment. In general, these observations suggest that several polysaccharide-specific PULs in *Sc*DSM18205 are rapidly co-upregulated upon encountering the polysaccharide mixture and that select PULs (associated with xylan, xyloglucan, and pectin backbones) are reactivated as carbon becomes limited.

**Fig 6:**
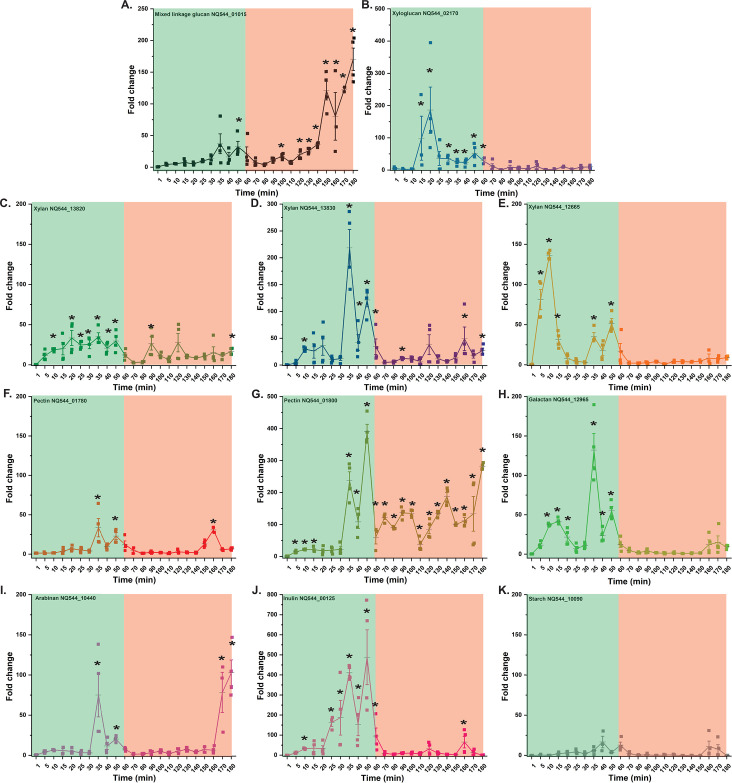
Temporal expression of *tbdt* transcripts of *Sc*DSM18205 in mYCFA supplemented with polysaccharide mixture (PM). (**A-K**) Temporal expression of individual *tbdt* was measured by RT-qPCR during growth on a mixture of eight polysaccharides that represent common complex plant-based dietary carbohydrates. All transcript level changes are relative to time zero prior to exposure to the PM. Error bars represent the SEM of two biological and two technical replicates. Asterisks indicate data points with upregulation significantly greater than a basal level (set at 10-fold), as determined by an unpaired *t* test. Background colors delineate early exponential (green) and exponential (salmon) growth phases.

Based on our previous observation of upregulation of *tbdts* on individual β-glucans in the late-lag/early-exponential phase (Fig. 4), the lack of an obvious lag phase on the polysaccharide mixture (Fig. 5A), and the apparent rapid upregulation of several PULs at the first sampling time (Fig. 5, *t* = 1 h), we repeated the RT-qPCR analysis with finer sampling over a 3 h period (Fig. 6). As before, the *tbdt* (NQ544_10090) from the degenerate *Sc*DSM18205 Sus (Fig. 3) was not upregulated. Within the 60-min period not captured in the previous experiment, we observed transient upregulation of several PULs at much higher levels (100-fold to 500-fold). Rapid sampling (every 5 min until *t* = 40 min, every 10 min thereafter) revealed temporal differences in the onset and termination of upregulation, which, in some cases, was rapid (e.g., xyloglucan-associated NQ544_02170 and pectic-arabinan-associated NQ544_10440). Consistent with the ready accessibility of short polysaccharides/oligosaccharides in the corn preparation, xylan-associated *tbdt* genes, especially NQ544_12665, were among those rapidly upregulated (Fig. 6 cf. Fig. 1). On the other hand, inulin likewise contains β-fructan chains of lower degrees of polymerization (72), and the associated *tbdt* (NQ544_00125) was the among the most highly upregulated (400-fold). Especially in the case of inulin, such levels were not observed in log- and stationary-phase time points (maximum 10-fold upregulation, Fig. 5), suggesting that this substrate is rapidly degraded. A similar situation may exist for xyloglucan (NQ544_02170), pectic galactan (NQ544_12965), pectic arabinan (NQ544_10440), and some xylan components (NQ544_12665) (Fig. 4 cf*.* Fig. 5). On the other hand, the utilization of mixed-linkage β-glucan appears to be deprioritized, with the associated *tbdt* (NQ544_01015) being upregulated much later. Finally, the data also suggest that although xylan- and pectin-backbone-associated *tbdt* genes are upregulated early, sufficient fractions of each polysaccharide apparently remain to trigger relatively high upregulation in the stationary phase (Fig. 4 cf*.* Fig. 5).

Thus, it is difficult to establish a clear order of polysaccharide prioritization by *Sc*DSM18205, which is likely complicated by several factors including the polydisperse nature of the polysaccharide preparation (with respect to molar mass and composition) and the compressed growth profile (lacking a lag phase and with rapid exponential growth) due to the use of the optimized mYCFA medium. Indeed, we did not observe any semblance of a diauxic (or multiauxic) growth pattern with the polysaccharide, as has been seen previously in *Bacteroides* species with longer culture durations (45, 46, 87). However, these data also suggest that *Sc*DSM18205 rapidly upregulates many PULs simultaneously in the early-exponential phase, thereby producing sufficient amounts of proteins and enzymes at that point to persist throughout the exponential phase, similar to what was observed with pure β-glucans (Fig. 4; Fig. S5). We also hypothesize that only after the easily accessed polysaccharides are depleted, does the bacterium reactivate PULs to address more persistent fractions.

### Coexistence of *Sc*DSM18205 and *Bacteroides* species

**Fig 7:**
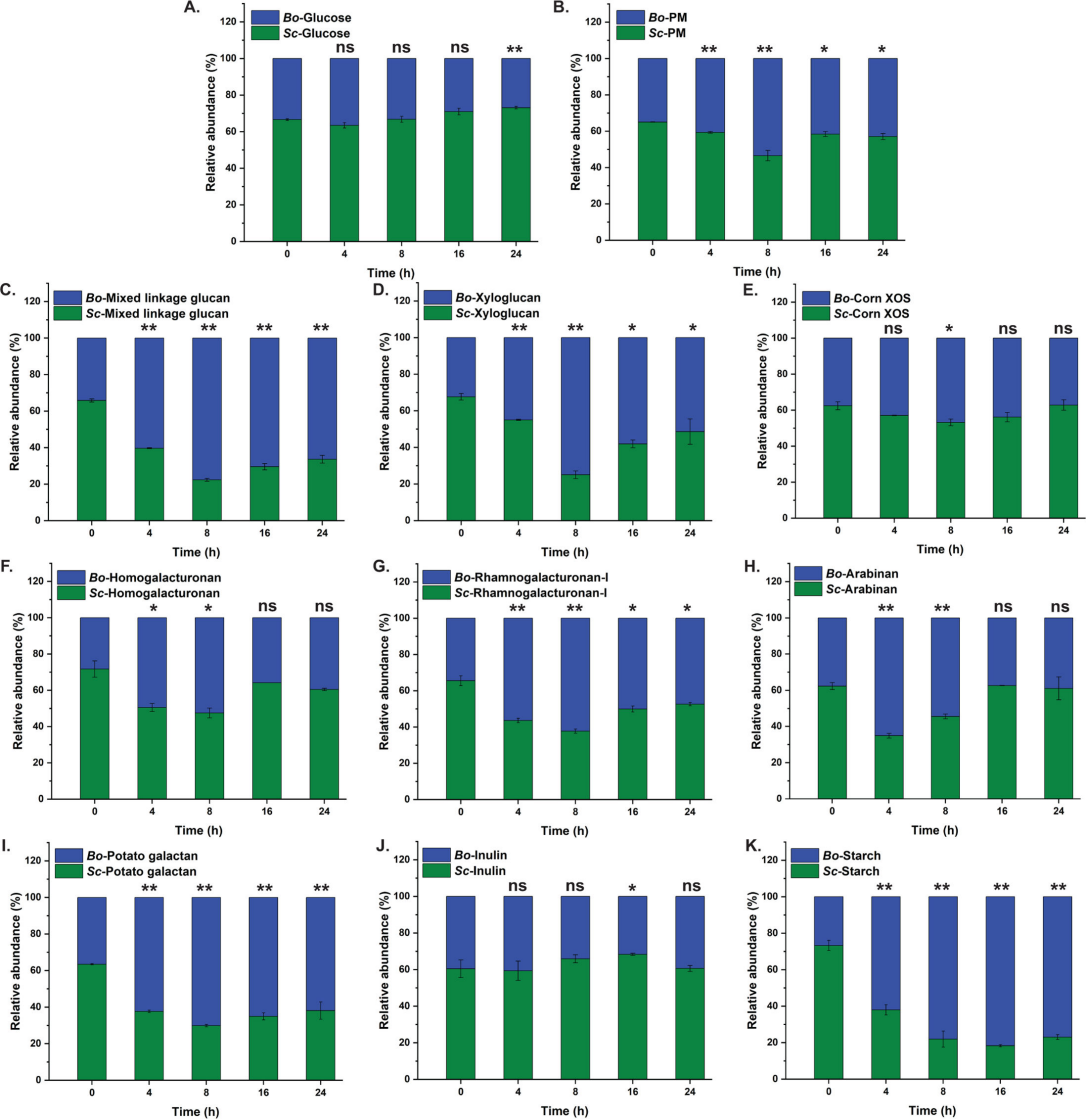
Relative abundance of *Sc*DSM18205 (*Sc*) and *B. ovatus* (*Bo*) co-cultured on defined carbohydrate sources in mYCFA. Abundances of each species on different polysaccharides were calculated using RT-qPCR with species-specific 16 s rRNA gene primers. Error bars represent the SD of the means of two biological replicates. Statistically significant differences were calculated using a two-tailed unpaired Student’s t test. *, *P* < 0.05; **, *P* < 0.01; ns, not significant (*P* > 0.05).

**Fig 8:**
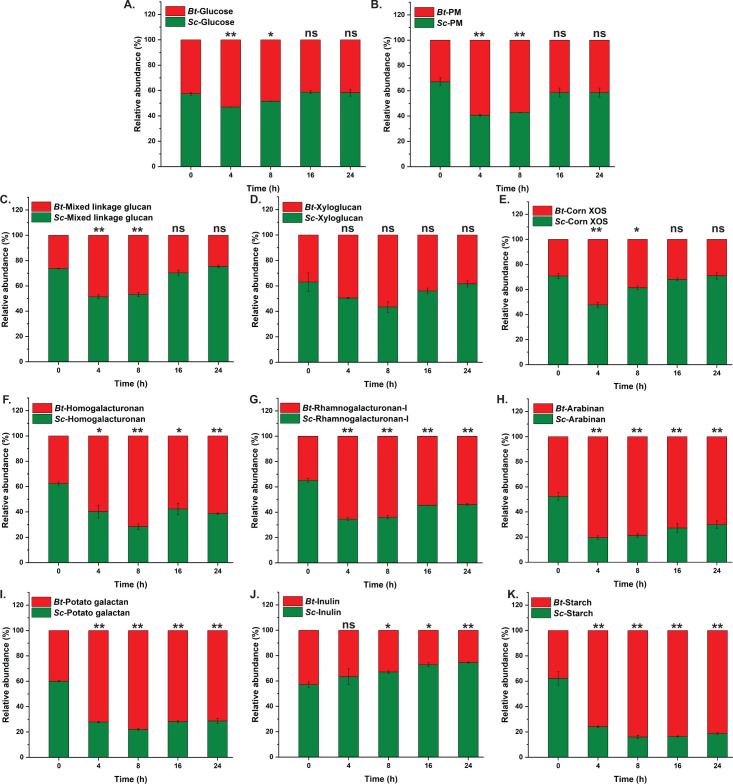
Relative abundance of *Sc*DSM18205 (*Sc*) and *B. thetaiotaomicron* (*Bt*) co-cultured on defined carbohydrate sources in mYCFA. Abundances of each species on different polysaccharides were calculated using RT-qPCR with species-specific 16 s rRNA gene primers. Error bars represent the SD of the means of two biological replicates. Statistically significant differences were calculated using a two-tailed unpaired Student’s t test. *, *P* < 0.05; **, *P* < 0.01; ns, not significant (*P* > 0.05).

The data above provided us with a solid understanding of the identity and deployment of individual polysaccharide utilization systems in *Sc*DSM18205. Building upon this foundational knowledge, we were then interested in exploring whether competition arising from differential utilization of plant polysaccharides might rationalize the reciprocal correlation of *Prevotellaceae* and *Bacteroidaceae* in fiber-rich and fiber-poor human diets. We selected *B. ovatus* ATCC8483 and *B. thetaiotaomicron* VPI-5482 as well-studied, representative *Bacteroidaceae* with distinct polysaccharide utilization profiles (44, 89) for pairwise competitive growth experiments with *Sc*DSM18205 *in vitro* (Fig. 7 and 8). To enable these studies, we first demonstrated that the polysaccharide growth profiles of *B. ovatus* ATCC8483 and *B. thetaiotaomicron* VPI-5482 in mYCFA media recapitulated those in standard media (44) (data not shown).

Reference points for noncompetitive and competitive growth were established with reference to growth on glucose and starch, respectively. *Sc*DSM18205, *B. ovatus*, and *B. thetaiotaomicron* individually grow well on glucose, and indeed, no significant change in relative abundance *versus* initial inocula is observed in extended co-cultures of *Sc*DSM18205 with *B. ovatus* (Fig. 7A) and *Sc*DSM18205 with *B. thetaiotaomicron* (Fig. 8A). In contrast, *Sc*DSM18205 is unable to utilize starch (Fig. S4; Table S3) due to a degenerate Sus (Fig. 3) and is rapidly out-competed by *B. ovatus* (Fig. 7K) or *B. thetaiotaomicron* (Fig. 8K), both of which grow readily on this ubiquitous dietary polysaccharide (Table S3, see also references [44, 90]). In the absence of other plant polysaccharides, for example, in a diet low in non-starchy fruits and vegetables (e.g., a starch-rich “Western” diet), this deficiency may already disadvantage *Sc*DSM18205 and other *Prevotellaceae* that lack viable starch utilization systems. Inulin represents another interesting reference: *B. ovatus*, but not *B. thetaiotaomicron*, is able to grow on this storage polysaccharide (Table S3) due to a specific β(1, 2)-fructan utilization locus (72). Hence, *Sc*DSM18205 and *B. ovatus* form a stable co-culture (Fig. 7J), whereas *Sc*DSM18205 dominates over *B. thetaiotaomicron* (Fig. 8J).

In most other cases, including with hemicelluloses, pectic polysaccharides, and the polysaccharide mixture, we observed that *Sc*DSM18205 is outcompeted by both *B. ovatus* and *B. thetaiotaomicron* in earlier time points but then rebounds to similar proportions as the initial inoculum (Fig. 7 and 8). The biochemical and biological bases for these kinetics are undoubtedly complex, but some cases deserve special mention. *B. thetaiotaomicron* lacks xyloglucan and mixed-linkage β-glucan utilization loci (44) (like *Sc*DSM18205, *B. ovatus* has both (50, 55)), yet briefly expands its population and is not completely dominated by *Sc*DSM18205 in co-culture. This suggests a potential for cross-feeding (47) of *B. thetaiotaomicron* by polysaccharide breakdown products from *Sc*DSM18205 (Fig. S7). Similar growth profiles are observed for xyloglucan and mixed-linkage β-glucan in *Sc*DSM18205/*B. ovatus* co-cultures. Growth rates for both bacteria on these individual polysaccharides are comparable (Table S3). In the case of mixed-linkage β-glucan, the rapid dominance by *B. ovatus* and the slow recovery of *Sc*DSM18205 may reflect the different catalytic machinery of the corresponding PULs in these bacteria, which use distinct endo-glucanses from different GH families (GH16 and GH5_4, respectively (50, 52)).

Likewise, initial dominance of the *Bacteroides* over *Sc*DSM18205 on pectic polysaccharides is not readily rationalized by examination of individual growth rates in monoculture (Table S3) but may be due to differences in the total protein systems encoded by their respective PULs. For example, *B. thetaiotaomicron* and *B. ovatus* have large repertoires of polysaccharide lyases in their pectic-associated PULs (40, 70) versus only two in the entire *Sc*DSM18205 genome. Factors such as protein abundance, enzyme efficiency, and transport kinetics will also be crucial factors.

Furthermore, disentangling the effects of individual pectin substructures is complicated by the difficulty in obtaining homogenous fractions, especially from commercial vendors. For example, *B. ovatus* initially dominates over *Sc*DSM18205 during growth on pectic arabinan (Fig. 7H), despite lacking a homologous arabinan-PUL (46; cf. Fig. 2). However, *B. ovatus* does grow on arabinose (Fig. S9A), and indeed, the monosaccharide accumulates in the medium during co-culture (Fig. S9B), potentially suggesting cross-feeding. *B. ovatus* also appears to grow weakly on pectic arabinan, despite the lack of a corresponding PUL, as does a strain in which we deleted genes for arabinose catabolism (Bovatus_00207–00211). Moreover, the competitiveness of this mutant is unaltered versus the wild-type (Fig. S9C). Further analysis by HPLC revealed the appearance of galactose in the medium of the original co-culture of *Sc*DSM18205 with wild-type *B. ovatus* (Fig. S9D). Altogether, these data indicate that *B. ovatus* was utilizing other polysaccharide impurities in the commercial pectic arabinan preparation, including 20% galactan (Megazyme product data).

## DISCUSSION

In the present study, we have extended earlier bioinformatic studies of HGM *Segatella* species by experimentally defining, in the type-strain *Sc*DSM18205, key PULs enabling the catabolism of ubiquitous plant polysaccharides. As exemplified by the *Sc*DSM18205 xyloglucan utilization system (Fig. 1), mRNA-seq is critical to resolve PUL and CAZymes Cluster boundaries, especially in those cases where abutting but non-correlated CAZymes are observed. Understanding genomic co-localization and co-regulation is essential to understanding the metabolic functions of individual CAZymes, especially those from polyspecific families (51, 91–93). The *Sc*DSM18205 PULs comprise a reference set to inform future comparative analyses of *Prevotellaceae* from both humans and other animals (41, 43, 63, 64, 94). For example, we note a direct concordance of PULs in *Sc*DSM18205 with those in rumen-associated species, which have likewise been defined by comprehensive transcriptomics, for example, the *S. bryantii* TF1-3 xyloglucan PUL and CAZyme cluster (94), the *Xylanibacter ruminicola* KHP1 mixed-linkage β-glucan PUL (94), and two *S*. *bryantii* B_1_4 xylan PULs (64). As we continue to uncover the molecular basis for dietary fiber utilization in the *Prevotellaceae*, it is becoming clear that there are remarkable similarities between monogastric and ruminant microbiota, despite the anatomical differences of their hosts (95) (for examples in the Bacillota, see references [96, 97]).

In comparison with previous studies on *Bacteroides* spp. (45, 46), we were surprised to observe very brief, transient upregulation of PULs upon initial exposure to individual plant polysaccharides or a mixture, just at the initiation of exponential-phase growth *in vitro* (Fig. 4 to 6). Although rapid upregulation of PULs in Bacteroidota is a common phenomenon, this response is often extended throughout growth (45, 46). Consequently, in a complex mixture meant to represent a selection of common dietary plant polysaccharides, we observed a bimodal regulation of several *Sc*DSM18205 PULs, in which a transient spike in transcription was followed by a return to basal levels and finally secondary upregulation in response to residual polysaccharides from the mixture (Fig. 5 and 6). The complexity of this response makes it difficult to unambiguously establish whether *Sc*DSM18205 has an inherent “preference” for individual polysaccharides, for example, in a complex digesta in the gut.

Moreover, it must be acknowledged that the sharp transition from culture on the pure monosaccharide glucose to complex polysaccharides on a timescale of minutes is quite artificial compared with the natural transit of digesta to the large intestine. Nonetheless, these *in vitro* data reveal that relief of glucose catabolite repression results in rapid production of manifold proteins from diverse PULs, enabling the rapid sensing and acquisition of polysaccharides in the immediate environment through their breakdown products (88). Interestingly, *Sc*DSM18205 appears to be a “sharing” (as opposed to “selfish”) member of the HGM (47, 73): Rapid upregulation of PULs, for example, those of mixed-linked β-glucan and xyloglucan (Fig. 4), results in a production of enzyme activity that obviously exceeds oligosaccharide transport kinetics, resulting in loss of soluble polysaccharide breakdown products to the medium (Fig. S7). Whether this would also be the case for cells operating on solid digesta particles in the gut may be an interesting topic of future study.

Considering the dichotomy of *Segatella* (*Prevotellaceae*) and *Bacteroides* (*Bacteroidaceae*), in pre-industrial *versus* post-industrial HGM, we observed no indication *in vitro* that common species in these genera cannot co-exist—at least on a suitably balanced mixture of plant polysaccharides. That is, *Sc*DSM18205 could generally be stably co-cultured with *B. ovatus* or *B. thetaiotaomicron* on individual plant polysaccharides, except where one was lacking a corresponding PUL. Another example was in the case of pectin utilization, where *B. thetaiotaomicron* outcompeted *Sc*DSM18205, perhaps due much more elaborate series of PULs in the former (70). Indeed, we recently observed that mixed-linkage β-glucan PULs from both *Segatella* and *Bacteroides* species were carried simultaneously in individual humans from diverse nations (52), which runs counter to the suggestion that these genera are mutually exclusive in HGM (see Reference (26) for a detailed discussion).

Most notably, *Sc*DSM18205 apparently lacks a complete starch utilization system (starch-PUL, Fig. 3), fails to grow on this common storage polysaccharide, and is therefore outcompeted by both *Bacteroides* species (Fig. 7 and 8). A survey of the PULDB (40) indicated that all currently annotated *Segatella* (as of August 2024) likewise lack cell-surface amylases (starch backbone hydrolases), as found in *B. ovatus* (GH13_47 (90) and *B. thetaiotaomicron* (GH13_36 (98) (Fig. 3). Additionally, resistant starch metabolism is particularly important for some species of Bacillota in the HGM, which constitute additional competitors (76).

*Segatella* species also generally lack PULs for the utilization of animal glycans, such as heparin, chondroitin sulfate, and mucin O-glycans, which are common in *Bacteroides* (44–46, 87). Very recent work in gnotobiotic mice has demonstrated that a human gut *S. copri* strain (H-2477) is quickly displaced by *Bacteroides* species in the absence of complex plant polysaccharides (27). Taken together, these observations suggest that two fundamental inequities conspire against *S. copri* in a post-industrial diet: an inability to utilize the predominant meat and starch polysaccharides (e.g., classic “steak and potatoes” or “burger and fries”), and the inability to forage on secreted host mucin as a survival strategy. We also note that the inability of *Sc*DSM18205 to grow on mannans appears to be due to an incomplete cognate PUL, as we characterized in the ruminant species *S. bryantii* B_1_4 (99), parts of which are found next to the xyloglucan PUL (Fig. 1, cf. Predicted PUL7 in the PULDB (40)).

Here, we have focused on metabolic inputs (catabolism); however, it is important to consider that individual taxa in the HGM have different metabolic outputs (anabolism), which will influence the host. As recent results show (30–32), our increasing understanding of the metabolic capacity and plasticity of glycan utilization in individual members of gut microbiota has tremendous potential to advance microbial or dietary interventions to improve health (100). The experimental definition of PULs and CAZyme clusters in *S. copri* DSM18205, together with co-growth data, in the present study will help guide future fundamental and applied research into the complex and dynamic HGM.

## MATERIALS AND METHODS

### Carbohydrates

Barley mixed-linkage β-glucan (P-BGBL), tamarind xyloglucan (P-XYGLN), wheat arabinoxylan (P-WAXYL), beechwood xylan (P-XYLNBE), citrus pectin polygalacturonic acid (homogalacturonan, P-PGACIT), potato galactan (P-GALPOT), pectic galactan (P-PGAPT), potato rhamnogalacturonan-I (P-RHAM1), sugar beet arabinan (P-ARAB), yeast β-glucan (P-BGYST), curdlan (P-CURDL), konjac glucomannan (P-GLCML), carob galactomannan (P-GALMH), and maltohexaose (O-MAL6) were from Megazyme. Gum Arabic arabinogalactan (G9752-500G), laminarin (L9634-1G), chicory inulin (I2255), amylose (A0512-1G), amylopectin (10120–250G), dextran (31389), pullulan (P4516-1G), glycogen (G0885-5G), maltose (M5885-100G), maltopentaose (M-8128), lactose (17814–1KG), galactose (G0750-100G), rhamnose (83650–10G), mannose (M8574), fructose (F0127-100G), ribose (R1757), galacturonic acid (48280–5G-F), and glucuronic acid (G5269) were from Sigma. Larch arabinogalactan (YL29121), corn xylan oligosaccharides (YC05381), sucrose (OS02339), raffinose (OR06197), melibiose (OM06702), and arabinose (MA02043) were from Carbosynth. Lentinan (TRC-L329100-5G) was from TRC, soluble starch (S516-100) from Fisher Scientific, glucose (BDH9230-2.5KG) from VWR, and cellobiose (108460050) from Acros.

### Bacterial strains and growth media

*Segatella copri* (syn. *Prevotella copri*) DSM 18205 and *Bacteroides thetaiotaomicron* VPI-5482 (DSM 2079) were obtained from the Leibniz Institute DSMZ–German Collection of Microorganisms and Cell Cultures GmbH. *Bacteroides ovatus* ATCC 8483 and the *Δtdk* derivative strain (55) were kind gifts from Professor Eric Martens (University of Michigan, Ann Arbor). Bacteria were grown in an anaerobic chamber at 37 ˚C (Coy Laboratory Products Inc., Grass Lake, MI; Atmosphere: 90% N_2_, 5% CO_2_, and 5% H_2_). Modified peptone-yeast-glucose (mPYG) and modified peptone-yeast-minimal (mPYM) media were prepared as previously described (52). For short chain fatty acid analysis, mPYG was used with further modification: glucose was omitted, and yeast extract was replaced with yeast nitrogen base. Modified yeast-casitone-fatty acid (mYCFA) medium was prepared as described in Table S1. All media were filter sterilized and allowed to equilibrate in the chamber for at least 6 h before use.

### Growth of *S. copri* DSM18025 on individual carbohydrates and RNA sequencing (RNA-seq)

Growth was monitored at 600 nm in 3 mL tubes using a handheld absorbance reader (Biochrom WPA CO8000). RNA-seq was performed on biological triplicates, using cells harvested at the mid-exponential phase (OD_600_ = 0.6–0.8), as detailed in (52). Raw reads were deposited under BioProject Accession PRJNA1073563 (Sequence Read Archive, National Center for Biotechnology Information). Reads were assessed using FastQC (101) and MultiQC (102), trimmed with Trimmomatic (103), aligned to the most recent *S. copri* DSM18025 genome (104), and counted using Salmon (105). Differential expression of each polysaccharide growth compared to glucose-grown cells was calculated using DESeq2 (106). PUL annotation was based on predicted PULs as defined in the PULDB (40). Predicted gene products were analyzed for signal peptides using SignalP (107).

### Growth of *S. copri* DSM18025 on carbohydrates in mYCFA

*Sc*DSM18205*, B. ovatus,* and *B. thetaiotaomicron* were grown in mPYM overnight (18 h). The cells were pelleted, washed in 2× mYCFA, and resuspended to a final OD_600_ of 0.08. Thereafter, 100 µL of previously sterilized carbohydrates (10 g/L) were mixed with 100 µL of 2× mYCFA containing cells in a flat bottom 96-well plate resulting in a final volume of 200 µL (5 g/L each carbohydrate and OD_600_ of 0.04). Plates were sealed and kept in a microplate reader (Powerwave HT, Biotek Instruments, Winooski, VT), and OD_600_ was recorded every 15 min (shaking the plate for 10 s) for 48 h. The experiment was performed in three biological replicates for each carbohydrate.

### Transcriptional profiling of *tbdt* homologs by RT-qPCR

For transcript analysis, *Sc*DSM18205 was propagated as described above and resuspended to OD_600_ 0.5 in 2× mYCFA devoid of any carbohydrate, prior to the addition of individual carbohydrates (mixed-linkage β-glucan, xyloglucan, and glucose; final concentration 2.5 g/L) or a polysaccharide mixture (PM: starch, inulin, xylan, mixed-linkage glucan, xyloglucan, homogalacturonan, rhamnogalacturonan I, and pectic arabinan; final total concentration 5 g/L). One milliliter of samples was collected at defined intervals (see Results and Discussion). The samples were instantly flash-frozen in liquid N_2_ and stored at −70°C for later analysis. To determine growth-limiting concentrations of the PM, *Sc*DSM18205 was grown in final total concentrations of 0.1 to 8 g/L, under similar conditions to those described above.

Frozen cells were thawed and pelleted (10,000 × *g*, 5 min, 4°C), and the supernatant was discarded. One hundred microliters of RNase-free lysozyme solution (10 g/L) in Tris-EDTA buffer (50 mM Tris, 10 mM EDTA, pH 8) was added to the pellet and incubated at 37°C for 10 min. Total RNA was extracted using TRI reagent (Sigma), following the manufacturer’s instructions until phase separation. The aqueous phase containing RNA was treated using the Monarch RNA cleanup kit (NEB) according to the supplier’s instructions. Contaminating DNA was removed by incubating the RNA sample (1 µg) with DNase I (NEB) at 37°C for 10 min. cDNA was synthesized by reverse transcription using the iScript cDNA synthesis kit (Bio-Rad) according to the supplier’s protocol. *tbdt* gene expression was quantified using specific primers (Table S4) and SYBR green master-mix (Bio-Rad) in an ABI 7500 FAST real-time PCR system (Applied Biosystems) for 40 cycles (95°C, 3 min; 95°C, 15 s; 60°C, 1 min), which was followed with a melting curve (60-95°C with 1 °C/min rise) to determine amplicon purity. Total reaction mixture in each well (10 µL) consisted of cDNA (20 ng), primer (0.4 µL; 400 nM), 2× master mix (5 µL), and RNase-free water (3.2 µL). Transcript levels at each time point were normalized versus the *recA* gene, after checking expression stability with other reference genes (*gyrA*, *dnaB,* and *16 s rRNA*) using GeNorm (108). The transcript levels of all *tbdt* genes were normalized to those of cells washed with mYCFA before inoculation into the respective polysaccharide or polysaccharide mixture (time zero).

### Carbohydrate analysis

Analytical thin-layer chromatography (TLC) of culture supernatants was performed on silica gel 60 F₂₅₄ (20 cm × 20 cm plates, Millipore). TLC plates were developed using a mobile phase of butanol/acetic acid/water (2:1:1), and analytes were visualized by heating after dipping into 0.5% orcinol/10% sulfuric acid.

High-performance anionic-exchange chromatography with pulsed amperometric detection (HPAEC-PAD) of culture supernatants was performed on a Dionex ICS-6000 system; Chromeleon 7 was used for instrument control and data analysis. All samples were filtered with 0.22 µm membrane filter prior to injection of 10 µL via autosampler held at 4°C. The eluents were ultrapure H_2_O (solvent A), 1.0 M NaOH (solvent B), and 1.0 M NaOAc (solvent C).

Mixed-linkage β-glucan degradation products and related standards were analyzed using Dionex CarboPac PA200 columns (3 mm × 50 mm guard column in series with a 3 mm × 250 mm analytical column) with the following gradient: 0–15 min, 10% B and linear 1%–30% C; 15–15.1 min, linear 10%–50% B, and 30%–50% C; 15.1–16 min, exponential 50%–10% B and 50%–1% C; 16–20.1 min, 10% B and 1% C. Arabinan degradation products and related standards were analyzed using Dionex CarboPac PA1 columns (2 mm × 50 mm guard column in series with a 2 mm × 250 mm analytical column) with the following gradient: 0–10 min, 10% B and linear 0%–10% C; 10–25 min 10% B and linear 10%–30% C; 25–30 min, 10% B and linear 30-90% C; 30–31 min, 10% B and exponential 90%–0% C; 31–40 min, 10% B and 0% C.

### Short chain fatty acid analysis

SCFA were analyzed by gas chromatography-mass spectrometry (GC-MS) according to a previously published protocol (109) with slight modification. Briefly, 1 mL of growth medium was centrifuged, and the supernatant was collected. The supernatant was diluted appropriately with ultrapure water and acidified with 5 M HCl (50 µL), followed by the addition of diethyl ether (500 µL). The samples were vortexed and kept on ice for 5 min, followed by centrifugation (10,000 × *g*, 5 min, 4°C). The upper layer (diethyl ether containing SCFA) was transferred into another tube containing 200 mg of Na_2_SO_4_, and 500 µL of diethyl ether was added. One hundred microliters of this sample was then transferred to a gas chromatography sample vial containing a glass insert (250 µL), and 5 µL of *N,O*-bis(trimethylsilyl)trifluoroacetamide (BSTFA) was added. The vials were capped, vortexed, and kept at 37°C for 2 h to form trimethylsilyl derivatives of SCFA, followed by injection into the GC/MS.

GC/MS analysis was performed on a 7890B gas chromatograph/5977 mass selective detector with a HB-5 capillary column (30 m × 0.25 mm × 0.25 µm; Agilent Technologies, Santa Clara, CA, USA). Helium was used as carrier gas at 1 mL/min. One microliter of the sample and standard was injected with a split ratio of 10:1. The column temperature was varied as follows: 40°C, 0–2.85 min, 10 °C/min ramp to 100°C, held for 1 min, 30 °C/min ramp to 250°C, and held for 1 min. In addition, 70 eV was used for electron impact (EI) ionization. MS data were acquired in selected ion monitoring (SIM) mode to minimize interference from reagents, by scanning for signals corresponding to trimethylsilyl derivatives of SCFA (acetate, m/z 117; propionate, *m/z* 131; butyrate, *m/z* 145, valerate, *m/z* 159, and succinate, *m/z* 147). Peak areas were analyzed using MassHunter and quantified versus calibration curves (0–1 mM SCFA).

### Co-culture experiments

Prior to co-culture, each bacterium was inoculated in mPYG and cultured overnight. Cells were then washed with 2× mYCFA devoid of carbohydrates and inoculated (OD_600_ ~0.04 of each species) into mYCFA containing 0.5% individual carbohydrates or the polysaccharide mixture in biological duplicates. One milliliter of samples from co-cultures was harvested at defined time intervals (0, 4, 8, 16, and 24 h) and stored in −70°C. The cells were thawed and pelleted (12,000 × *g*, 5 min, 4°C), and in some cases, the supernatant was stored at −20°C for later use. Cells were first treated with lysozyme (10 g/L), followed by genomic DNA extraction using the gSYNC DNA Extraction Kit (Geneaid). The relative abundance of each species was determined by qPCR using specific primers (Table S4), and SYBR green master-mix reagent (Bio-Rad) in an ABI 7500 FAST real-time PCR instrument in a 96-well plate (Applied Biosystems). The total reaction mixture in each well (10 µL) was formulated by adding diluted gDNA (1 µL; 0.1 ng/µL), each primer (0.4 µL; 400 nM), master mix 2 x (5 µL), and sterile water (3.2 µL). A standard curve was obtained for the gDNA of each bacterial species at a range of concentrations (0.001, 0.01, 0.1, 1, 10, 20, and 40 ng/µL). The lack of cross-reactivity of each primer set was confirmed under the same conditions.

### *B. ovatus* gene deletion

In-frame deletion of arabinose catabolism genes was performed in the *B. ovatus Δtdk* strain background, essentially as described previously (55, 98). Briefly, 1 kb regions upstream and downstream of gene loci Bovatus_00207–00211 were cloned into the pExchange vector via Gibson assembly using primers listed in Table S5, according to the supplier’s instructions (New England Biolabs), and transformed into *Escherichia coli* S17.1 λ-pir. *B. ovatus Δtdk* was grown in TYG medium (110) and conjugated with the transformed *E. coli* strain, and the knockout was confirmed by PCR.

## Data Availability

Raw RNA sequencing reads generated during this study were deposited in the Sequence Read Archive, National Center for Biotechnology Information, under BioProject accession PRJNA1073563. The whole-genome sequence of *S. copri* DSM 18205 used for read mapping was obtained from GenBank accession CP102288.1.

## SUPPLEMENTAL MATERIAL

Supplemental material aem.01759-24-s0001.pdf Tables S1 to S5 and Figures S1 to S9.

ASM does not own the copyrights to Supplemental Material that may be linked to, or accessed through, an article. The authors have granted ASM a non-exclusive, world-wide license to publish the Supplemental Material files. Please contact the corresponding author directly for reuse.
