# Supplementary material for: Transcriptional delineation of polysaccharide utilization loci in the human gut commensal Segatella copri DSM18205 and co-culture with exemplar Bacteroides species on dietary plant glycans
Source: Appl Environ Microbiol. 2024 Dec 5;91(1):e01759-24. doi: 10.1128/aem.01759-24 (PMC11784079; doi:10.1128/aem.01759-24)
Supplement: Supplemental material — Tables S1 to S5 and Figures S1 to S9. [file aem.01759-24-s0001.pdf]

Supplemental Tables

Table S1. Modified Yeast Casitone Fatty Acid (mYCFA) medium composition (pH 7.2)

**mYCFA**	**2X- 50mL**
Trypticase peptone	0.25 g
Peptone	0.25 g
Yeast Nitrogen base	0.25 g
Tryptone	1.0 g
Cysteine hydrochloride	100 mg
Haemin (0.5 g/L)	2 mL
Salt solution (10X)	10 mL
Short chain fatty acid solution (100X)	1 mL
Vitamin Mix-1 (PYM)	100 μL
Vitamin Mix-2 (PYM)	100 μL
CaCl2 (100X, PYG)	1 mL
MgSO4 (100X, PYG)	1 mL

**Salt solution 10X**	**100 mL**
$NaHCO_3$	4 g
K_2HPO_4	0.45 g
KH_2PO_4	0.45 g
NaCl	90 mg

**SCFA solution (100X)**	**100 mL**
Acetic acid	3300 mM
Propionic acid	900 mM
Isobutyric acid	100 mM
Isovalaric acid	100 mM
Valaric acid	100 mM

Table S2. Growth parameters of *Segatella copri* DSM 18205, *Bacteroides ovatus* ATCC 8483, and *B. thetaiotaomicron* VPI-5482 on various substrates in mYCFA (multiwell plates).

Substrates	Growth rate / h		
	***Sc*DSM18205**	***Bo*ATCC8483**	***Bt*VPI-5482**
**Polysaccharides**			
Xyloglucan	0.095 ± 0.016	0.084 ± 0.001	-*
Mixed linkage glucan	0.080 ± 0.002	0.064 ± 0.001	-
Corn XOS	0.107 ± 0.004	0.062 ± 0.001	-
Wheat arabinoxylan	0.102 ± 0.008	0.050 ± 0.003	-
Yeast beta glucan	0.026 ± 0.001	0.036 ± 0.001	0.064 ± 0.001
Rhamnogalacturonan-I	0.043 ± 0.010	0.039 ± 0.001	0.129 ± 0.001
Homogalacturonan	0.082 ± 0.002	0.041 ± 0.001	0.125 ± 0.001
Arabinan	0.094 ± 0.005	0.018 ± 0.001	-
Potato galactan	0.098 ± 0.010	0.076 ± 0.001	0.149 ± 0.002
Inulin	0.120 ± 0.003	0.068 ± 0.002	-
Starch	n.d.**	0.074 ± 0.001	0.104 ± 0.003
**Oligosaccharides**			
Maltose	0.167 ± 0.001	0.141 ± 0.001	-
Raffinose	0.152 ± 0.002	0.151 ± 0.001	-
Cellobiose	0.042 ± 0.001	0.061 ± 0.001	-
Melibiose	0.143 ± 0.007	0.160 ± 0.001	-
Sucrose	0.128 ± 0.001	0.140 ± 0.001	-
Lactose	0.099 ± 0.005	0.111 ± 0.001	-
**Monosaccharides**			
Glucose	0.150 ± 0.003	0.164 ± 0.001	0.169 ± 0.004
Ribose	n.d.	0.119 ± 0.009	-
Mannose	n.d.	0.153 ± 0.005	-
Galactose	0.187 ± 0.003	0.155 ± 0.005	-
Galacturonic Acid	0.067 ± 0.002	0.037 ± 0.001	-
Arabinose	0.175 ± 0.004	0.117 ± 0.004	-
Fructose	0.150± 0.002	0.144 ± 0.002	-
Xylose	0.123 ± 0.006	0.118 ± 0.005	-
Rhamnose	0.027 ± 0.001	0.075 ± 0.006	-
Glucuronic Acid	0.034 ± 0.001	0.066 ± 0.001	-
No sugar	0.0017 ± 0.00029	0.0029 ± 0.00014	0.0014 ± 0.00012

* Not measured; lack of growth known from previous studies.
** n.d., not detected.

Table S3: Carbohydrate composition of substrates.

Substrates	Monosaccharide composition (%)
Tamarind xyloglucan[a]	Xylose: Glucose: Galactose: Arabinose: Other sugars = 34: 45: 17: 2: 2
Barley mixed linkage glucan[a]	Glucose = 97
Corn cob XOS[b]	Xylose substituted with acetyl, 4-O-methyl-D-glucuronosyl and α-1,3 L-arabinofuranosyl residues
Wheat arabinoxylan[a]	Arabinose: Xylose = 38: 62
Beechwood xylan[a]	Xylose: Glucuronic Acid: Other sugars = 84: 10.3: 5.7
Yeast β-glucan[a]	Glucose > 80%
Rhamnogalacturonan-I[a] (potato)	Galacturonic Acid: Rhamnose: Arabinose: Xylose: Galactose: Other Sugars = 61.0: 6.2: 2.5: 0.5: 23.1: 6.7
Citrus Homogalacturonan[a]	Galacturonic Acid: Rhamnose: Arabinose: Xylose: Galactose: Other Sugars = 61.0: 6.2: 2.5: 0.5: 23.1: 6.7
Sugar beet arabinan[a]	Arabinose: Galactose: Rhamnose: Galacturonic acid: Other sugars = 69: 18.7: 1.4: 10.2: 0.7
Galactan[a] (potato)	Galactose: Arabinose: Rhamnose: Galacturonic acid = 88: 3: 4: 5
Pectic galactan[a] (potato)	Galactose: Arabinose: Rhamnose: Galacturonic acid: Other sugars = 74: 0.1: 11.4: 10: 4.5
Chicory Inulin[c]	Glucose and Fructose
Starch soluble[d]	Glucose

[a]Megazyme product description
[b]Biosynth product description (composition not available)
[c]Sigma Aldrich product description (composition not available)
[d]Fisher Scientific product description (composition not available)

Table S4. RT-qPCR primers.

Primer Name	Sequence (5'→3')	Use
MLG_F	CGCTTGCTTACCCTCTATGC	MLG_*susC*-homolog expression
MLG_R	ATGCCTTTACGGTTGACAGG	MLG_*susC*-homolog expression
Pectin NQ_01800_F	ACCAAGGACAACATCAACGC	Pectin_*susC*-homolog expression
Pectin NQ_01800_R	TGGGCGGAATGTAACAGTCT	Pectin_*susC*-homolog expression
Pectin NQ_01780_F	TAACTTCTACCTGCGTGCCA	Pectin_*susC*-homolog expression
Pectin NQ_01780_R	TTGTCCCACGTCCAGTCTAC	Pectin_*susC*-homolog expression
PG_F	CGCTTGCTTACCCTCTATGC	Galactan_*susC*-homolog expression
PG_R	ATGCCTTTACGGTTGACAGG	Galactan_susC-homolog expression
Arab_F	TCTGCCGTTTCTACCTTCGT	Arabinan_*susC*-homolog expression
Arab_R	TCGAAGCCACTGTTCTCCAT	Arabinan_susC-homolog expression
XyG_F	AGAGAAGGGACATGCTGACC	Xyloglucan_*susC*-homolog expression
XyG_R	CAAAGTCCAACCGGCAGAAA	Xyloglucan_susC-homolog expression
Xy NQ_12655_F	ACTTGGGACGCTGGTATCAA	Xylan_*susC*-homolog expression
Xy NQ_12655_R	ACCTGGCTCAAGAATGTCGA	Xylan_*susC*-homolog expression
Xy NQ_13820_F	CCGCTCCATCCCATCTTACT	Xylan_*susC*-homolog expression
Xy NQ_13820_R	GCTGAGCGTCATCCTTCAAG	Xylan_*susC*-homolog expression
Xy NQ_13830_F	CTTCACATTCTTCGCCCTCG	Xylan_*susC*-homolog expression
Xy NQ_13830_R	TGTACCTTAGCCAAGCGGAA	Xylan_*susC*-homolog expression
Inu_F	AGACGTGGATTTTCGACCCT	Inulin_*susC*-homolog expression
Inu_R	GCTGTGTTCATGGTGCTCAA	Inulin_susC-homolog expression
ST_F	TGAAGCTGGAACTGGGCATA	Starch_*susC*-homolog expression
ST_R	TGATGTTGACCGTTGCCTTG	Starch_*susC*-homolog expression
Sc recA_F	GGTTCGCGTAAAGGTGGTAA	*recA* gene normalization expression
Sc recA_R	TATACCAGCTGCCGCTCTTT	*recA* gene normalization expression
Sc 16s_F	TTTGGAGACAATGACGCCCT	specific to *Sc* enumeration
Sc 16s_R	ACTTAAGCCGACACCTCACG	
Bo 16s_F	GTAAGGCTGTTGTGAAGG	specific to *Bo* enumeration
Bo 16s_R	TAGCTGCCTTCTGTACCC	
Bt 3854_F	CTTTATTCTGGTCCGGCAGC	specific to *Bt* enumeration
Bt 3854_R	TCATACGGGTCGGACAGATG	

Table S5. Primers for reverse genetics.

Primer Name	Sequence (5'→3')	Use
Bo_Ara_US_F	ATATCGAATTATGGAAGTATGCATCACTAAC	Upstream region
Bo_Ara_US_R	CTGTCAATTAAATAAATATGATTTATAAGTT AATATTATTTCTCTAC	Upstream region
Bo_Ara_DS_F	CATATTTATTTAATTGACAGTCGGCAATC	Downstream region
Bo_Ara_DS_R	GGGCTGCAGGAATTCTTGTCGTCTTGTG	Downstream region
Bo_Ara_ pEx_F	GACAAGAATTCCTGCAGCCCGGGGGATC	Vector region
Bo_Ara_pEx _R	ATACTTCCATAATTCGATATCAAGCTTATCGA TACCGTCGACTCG	Vector region

Supplemental Figures

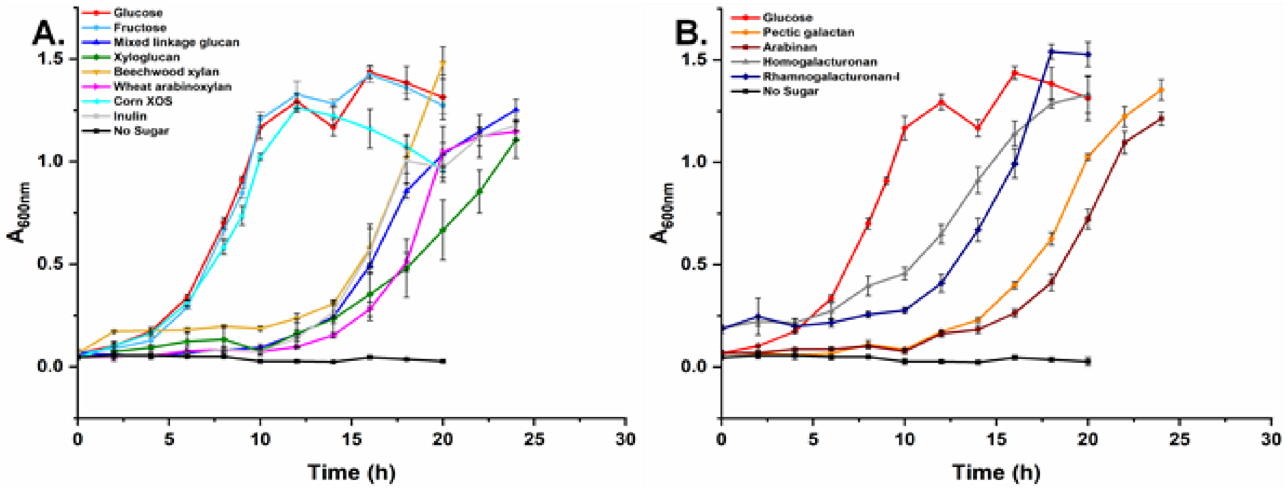

Figure S1. ***S. copri*** **DSM18205 growth in modified peptone-yeast media (mPYM) containing individual monosaccharides and polysaccharides (5 g/L).** Glucose and fructose were used as positive controls. **(A)** β-fructan, β-glucan, and β-xylan, chicory inulin; barley mixed linkage β-glucan; tamarind xyloglucan; beechwood glucuronoxylan; wheat arabinoxylan; corn arabinoxylan; **(B)** pectic polysaccharides: homogalacturonan; rhamnogalacturonan I; pectic galactan, arabinan. Error bars represent standard deviations (*n* =3 biological replicates).

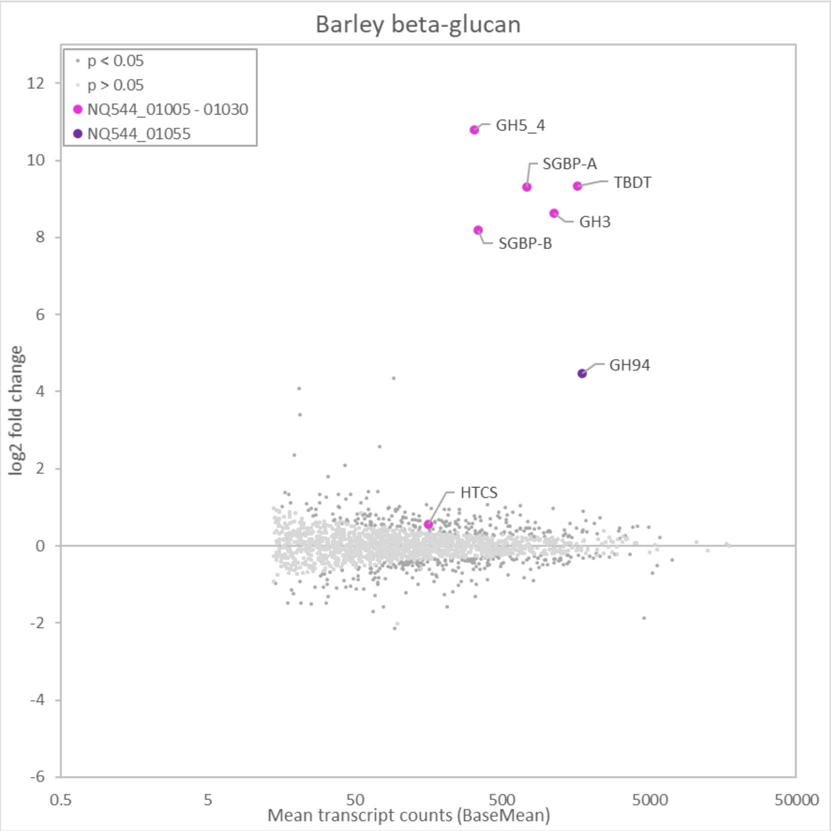
Barley beta-glucan
p < 0.05
p > 0.05
NQ544_01005 - 01030
NQ544_01055
GH5_4
SGBP-A
TBDT
GH3
SGBP-B
GH94
HTCS
log2 fold change
12
10
8
6
4
2
0
-2
-4
-6
0.5
5
50
500
5000
50000
Mean transcript counts (BaseMean)

A.

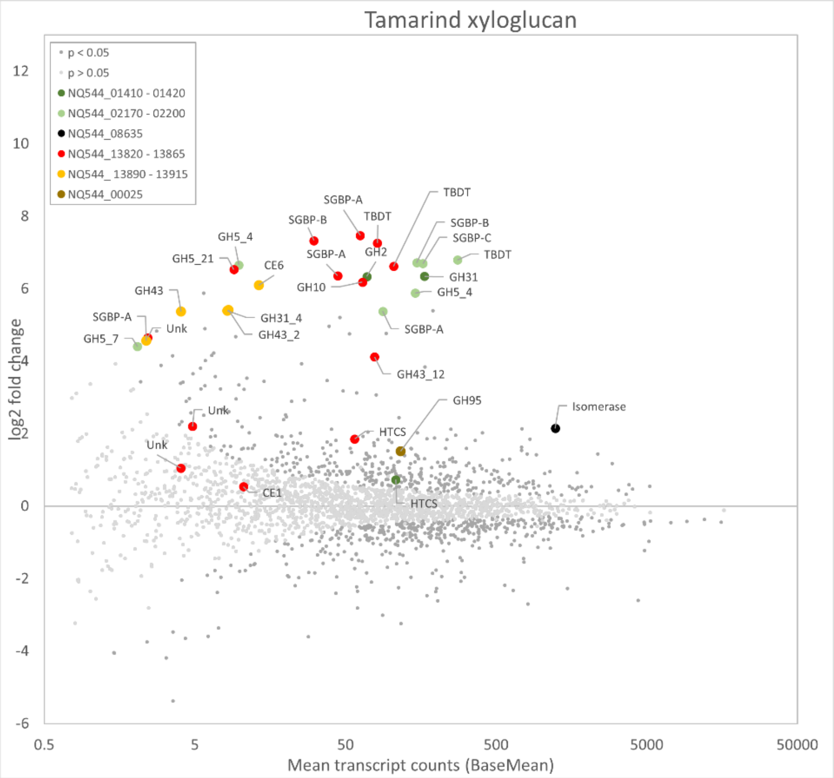
Tamarind xyloglucan
p < 0.05
p > 0.05
NQ544_01410 - 01420
NQ544_02170 - 02200
NQ544_08635
NQ544_13820 - 13865
NQ544_ 13890 - 13915
NQ544_00025
SGBP-A
SGBP-B
TBDT
TBDT
SGBP-B
SGBP-C
TBDT
GH5_4
GH5_21
CE6
SGBP-A
GH2
GH10
GH31
GH5_4
GH43
SGBP-A
GH31_4
GH43_2
SGBP-A
Unk
GH5_7
GH43_12
Unk
GH95
Isomerase
HTCS
Unk
CE1
HTCS
log2 fold change
12
10
8
6
4
2
0
-2
-4
-6
0.5
5
50
500
5000
50000
Mean transcript counts (BaseMean)

B.

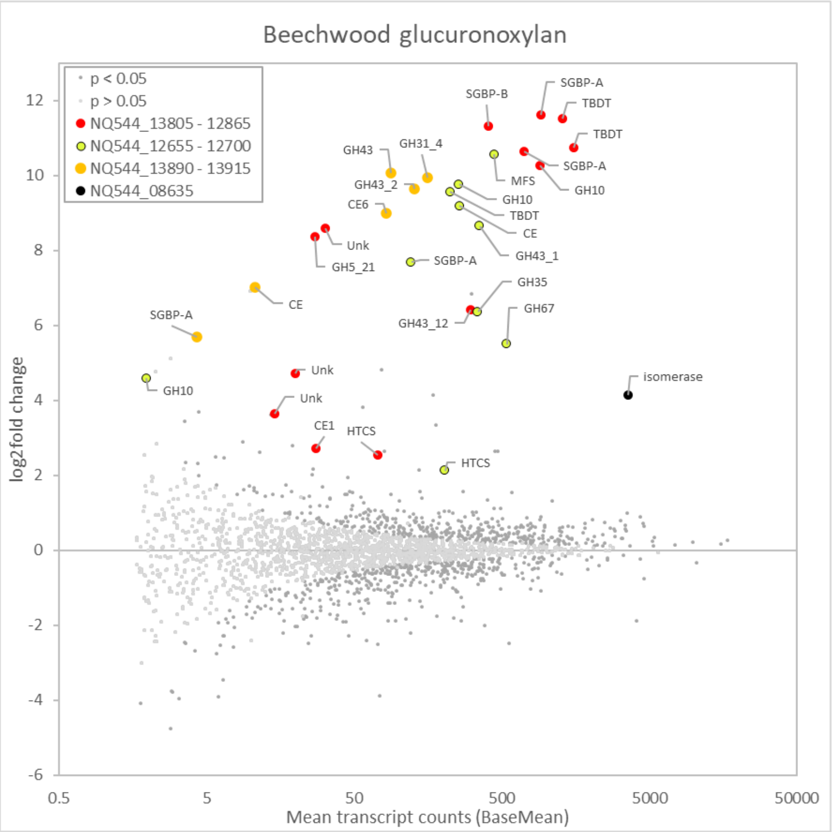
Beechwood glucuronoxylan
p < 0.05
p > 0.05
NQ544_13805 - 12865
NQ544_12655 - 12700
NQ544_13890 - 13915
NQ544_08635
SGBP-A
TBDT
SGBP-B
TBDT
GH43
GH31_4
SGBP-A
GH43_2
MFS
GH10
CE6
GH10
TBDT
CE
Unk
GH43_1
GH5_21
SGBP-A
GH35
CE
GH67
SGBP-A
GH43_12
isomerase
Unk
GH10
Unk
CE1
HTCS
HTCS
log2fold change
Mean transcript counts (BaseMean)
12
10
8
6
4
2
0
-2
-4
-6
0.5
5
50
500
5000
50000

C.

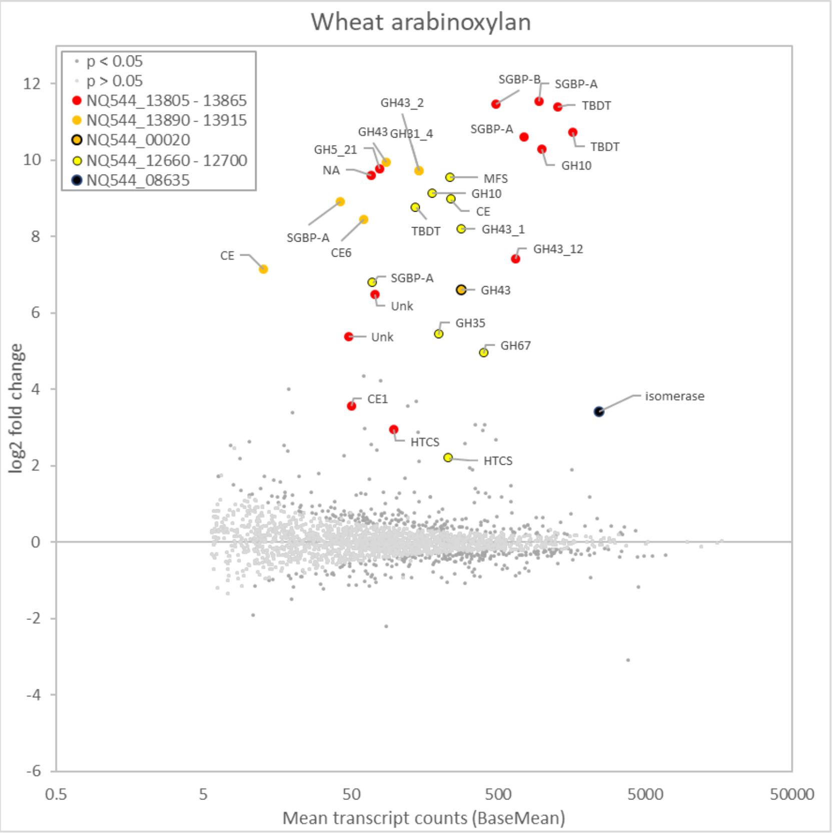
Wheat arabinoxylan
p < 0.05
p > 0.05
NQ544_13805 - 13865
NQ544_13890 - 13915
NQ544_00020
NQ544_12660 - 12700
NQ544_08635
SGBP-B
SGBP-A
GH43_2
TBDT
GH43
GH31_4
SGBP-A
TBDT
GH5_21
GH10
NA
MFS
GH10
CE
SGBP-A
TBDT
GH43_1
CE6
CE
GH43_12
SGBP-A
GH43
Unk
GH35
Unk
GH67
isomerase
CE1
HTCS
HTCS
log2 fold change
Mean transcript counts (BaseMean)
12
10
8
6
4
2
0
-2
-4
-6
0.5
5
50
500
5000
50000

D.

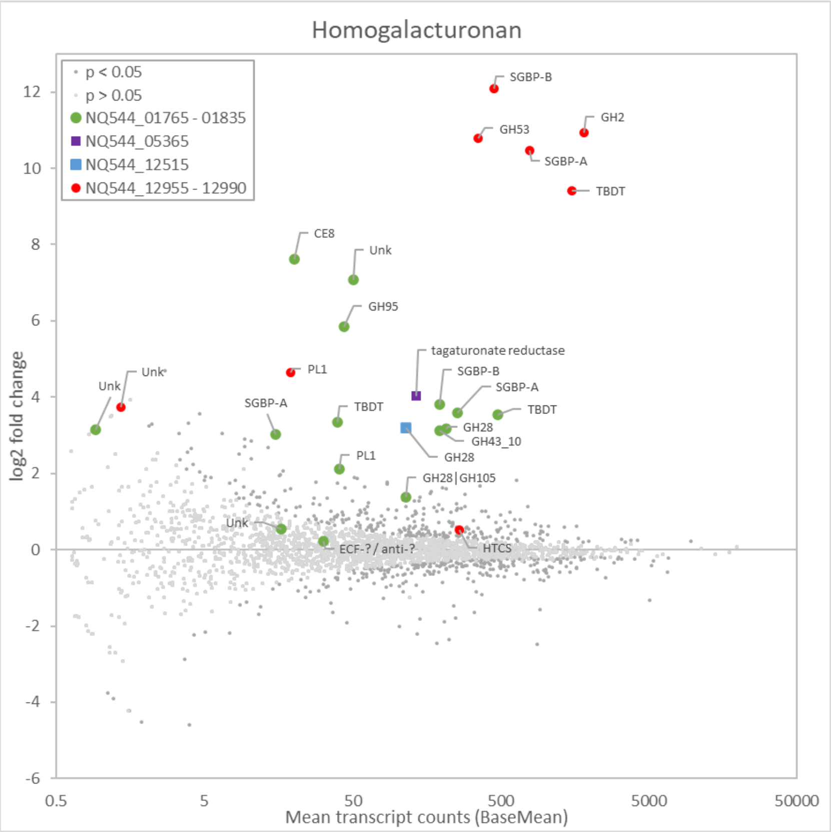
Homogalacturonan
p < 0.05
p > 0.05
NQ544_01765 - 01835
NQ544_05365
NQ544_12515
NQ544_12955 - 12990
SGBP-B
GH2
GH53
SGBP-A
TBDT
CE8
Unk
GH95
tagaturonate reductase
PL1
Unk
Unk
SGBP-B
SGBP-A
SGBP-A
TBDT
TBDT
GH28
GH43_10
GH28
PL1
GH28|GH105
Unk
ECF-? / anti-?
HTCS
log2 fold change
Mean transcript counts (BaseMean)
12
10
8
6
4
2
0
-2
-4
-6
0.5
5
50
500
5000
50000

E.

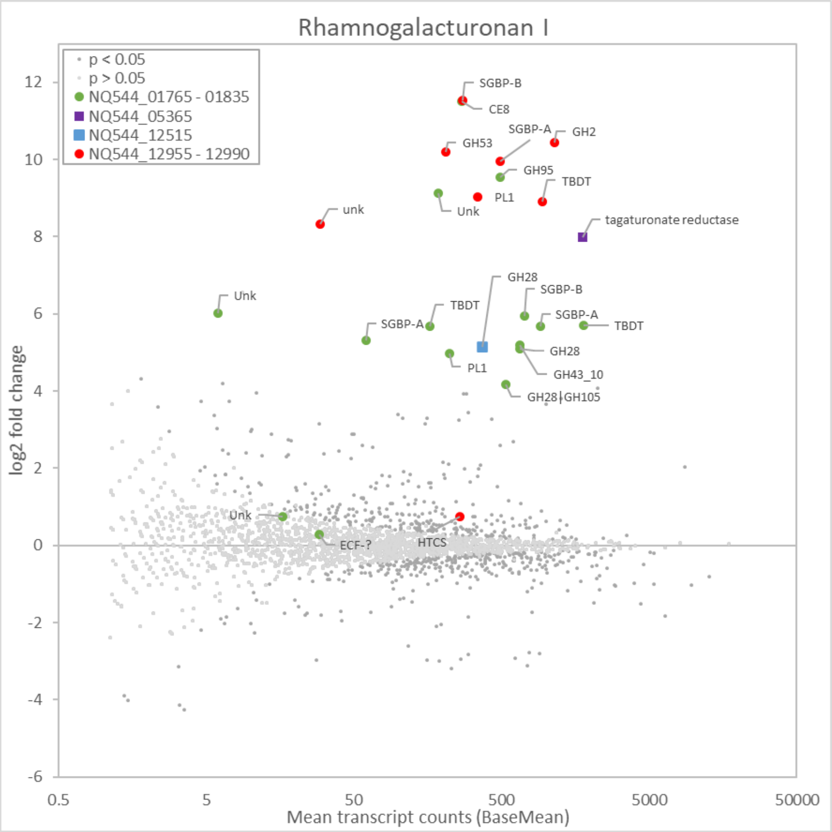
Rhamnogalacturonan I
p < 0.05
p > 0.05
NQ544_01765 - 01835
NQ544_05365
NQ544_12515
NQ544_12955 - 12990
SGBP-B
CE8
SGBP-A
GH2
GH53
GH95
TBDT
PL1
Unk
unk
tagaturonate reductase
GH28
SGBP-B
Unk
TBDT
SGBP-A
SGBP-A
TBDT
GH28
PL1
GH43_10
GH28|GH105
Unk
ECF-?
HTCS
log2 fold change
Mean transcript counts (BaseMean)
12
10
8
6
4
2
0
-2
-4
-6
0.5
5
50
500
5000
50000

F.

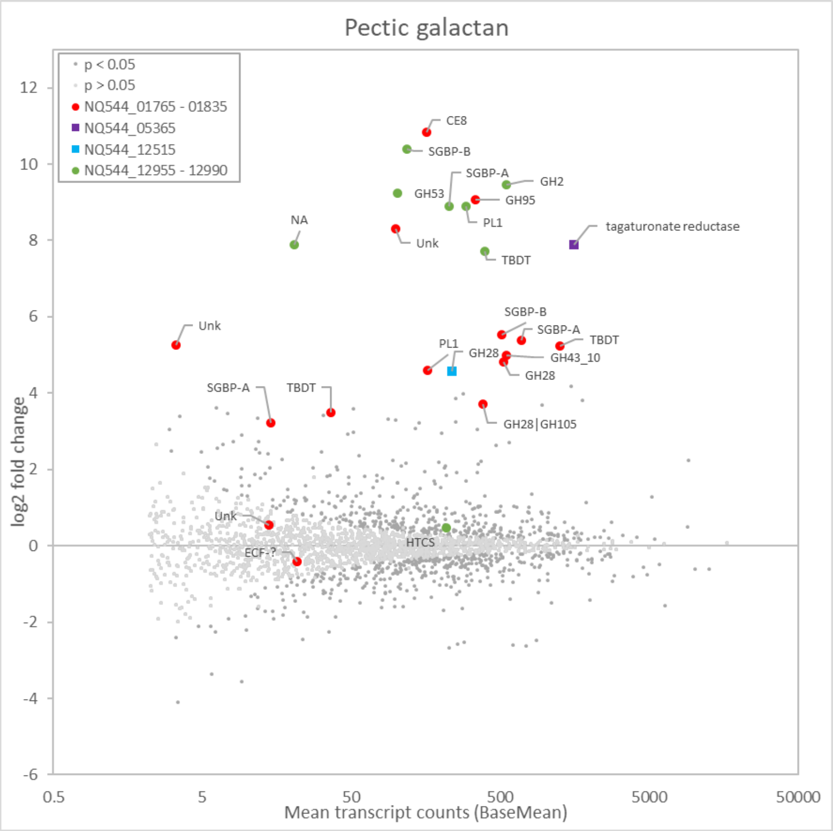
Pectic galactan
p < 0.05
p > 0.05
NQ544_01765 - 01835
NQ544_05365
NQ544_12515
NQ544_12955 - 12990
CE8
SGBP-B
SGBP-A
GH2
GH53
GH95
PL1
NA
Unk
tagaturonate reductase
TBDT
GH28
GH43_10
GH28|GH105
HTCS
ECF-?
log2 fold change
Mean transcript counts (BaseMean)

G.

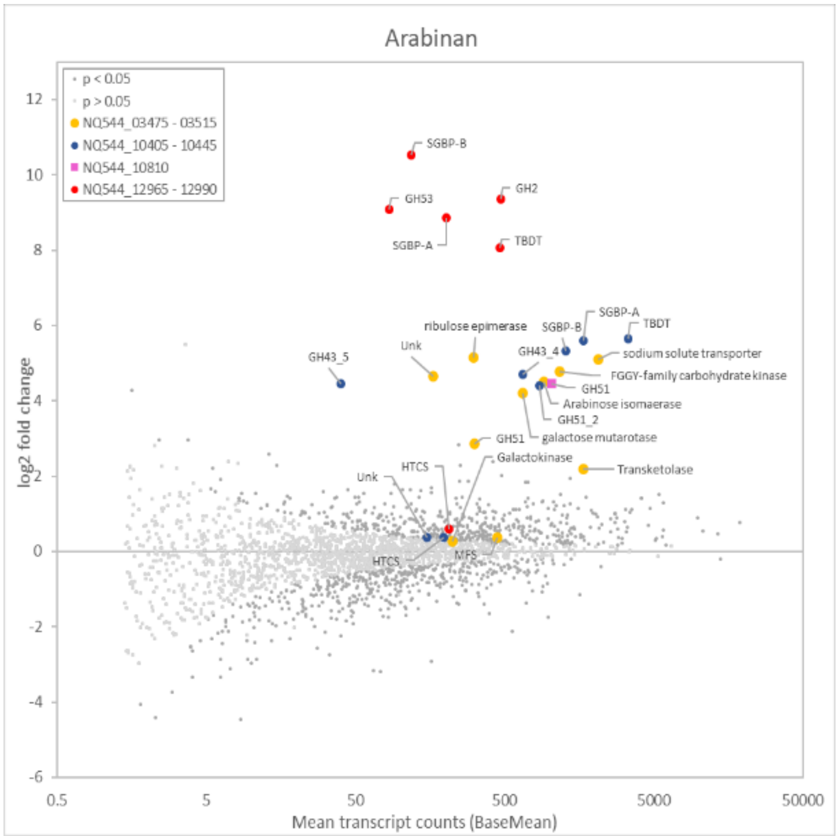
Arabinan
p < 0.05
p > 0.05
NQ544_03475 - 03515
NQ544_10405 - 10445
NQ544_10810
NQ544_12965 - 12990
SGBP-B
GH53
GH2
SGBP-A
TBDT
ribulose epimerase
Unk
GH43_5
GH43_4
sodium solute transporter
FGGY-family carbohydrate kinase
GH51
Arabinose isomaerase
GH51_2
galactose mutarotase
Galactokinase
HTCS
Transketolase
MFS
log2 fold change
Mean transcript counts (BaseMean)

H.

I.

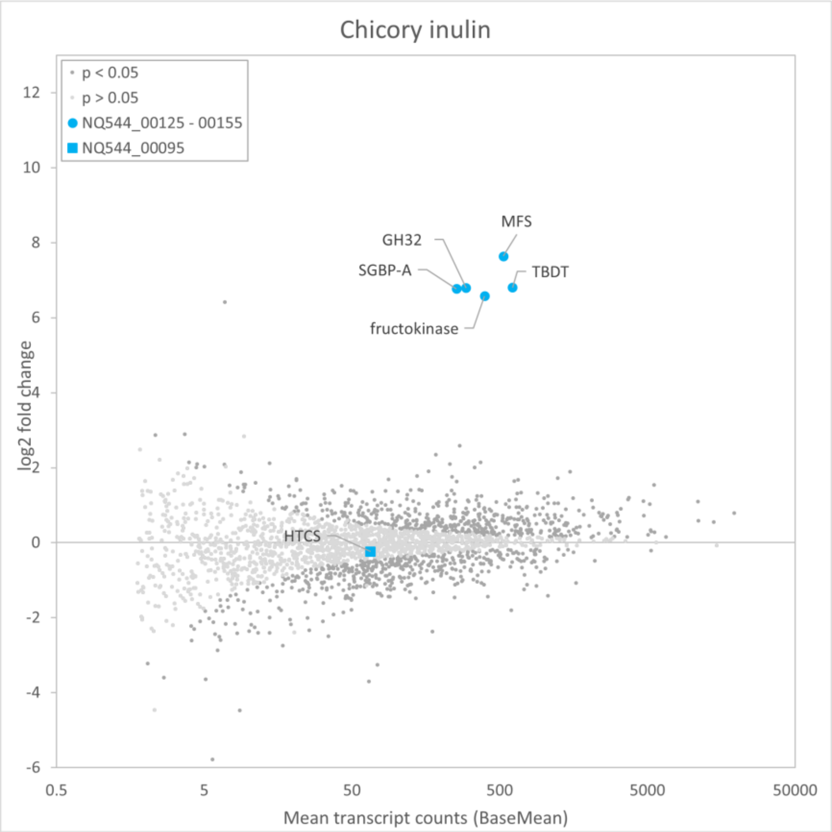

Figure S2. Differential expression of *S. copri* DSM18205 genes during growth on individual polysaccharides versus glucose. A.-I. MA plots with polysaccharides denoted in panel titles. Key genes encoding Glycoside Hydrolase (GH), Polysaccharide Lyase (PL), Surface Glycan Binding Protein (SGBP), TonB-Dependent Transporter (TBDT), Hybrid Two-Component System (HTCS), and Major Facilitator Superfamily (MFS) proteins are indicated.

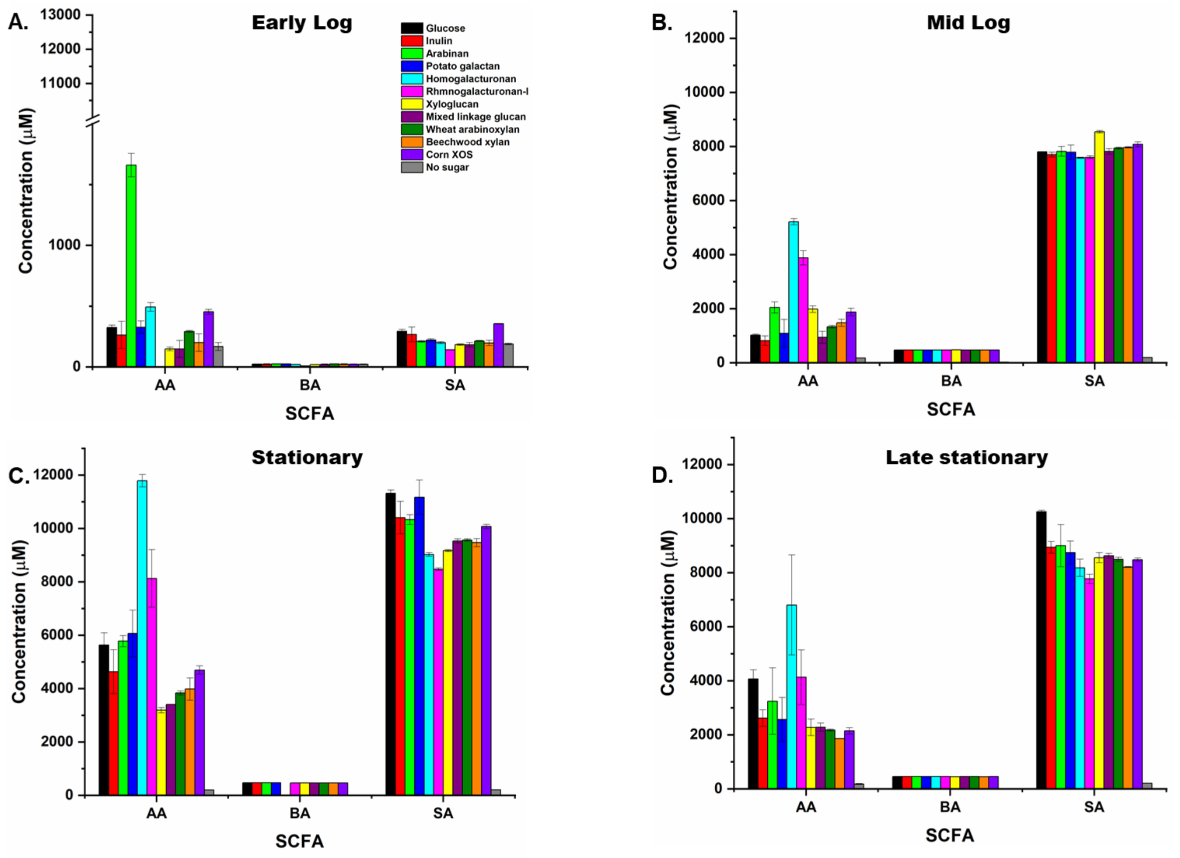

Figure S3. Short chain fatty acid production from plant polysaccharides by *Sc*DSM18205. SCFA profiling at different growth stages of *Sc* on various plant glycans where levels of acetic acid (AA), butyric acid (BA) and succinic acid (SA) monitored via GC-MS after BSTFA derivatization. **(A)** Early exponential phase (**B**) Mid exponential phase (**C**) Stationary, and (**D**) Late stationary phase. Error bars represent SD between two biological replicates.

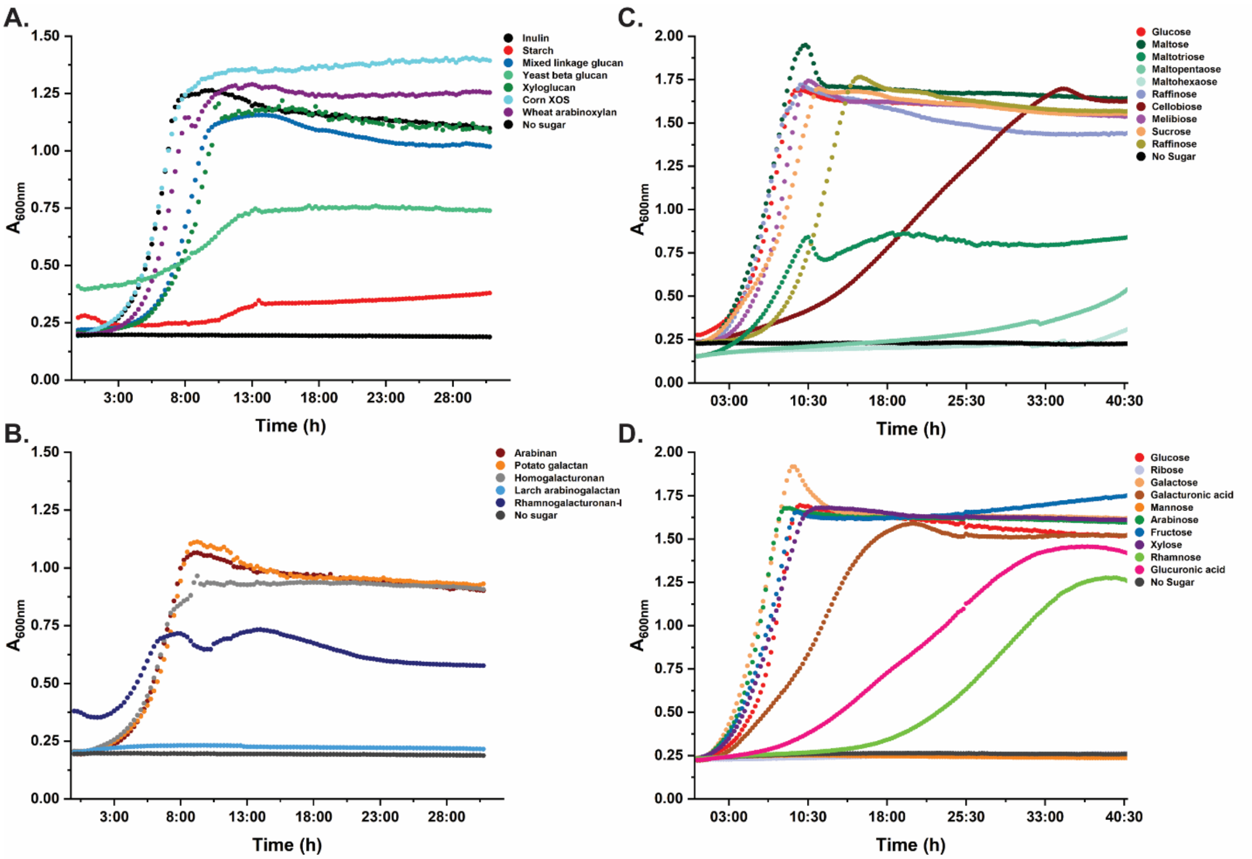

Figure S4. ***Sc*DSM18205 growth kinetics on mYCFA supplemented with different substrates (5 g/L) as the sole carbohydrate source. (A)** Hemicelluloses, inulin, and starch. **(B)** pectic polysaccharides. **(C)** oligosaccharides. **(D)** monosaccharides. Data are mean values of three independent biological replicates.

Mixed linkage glucan

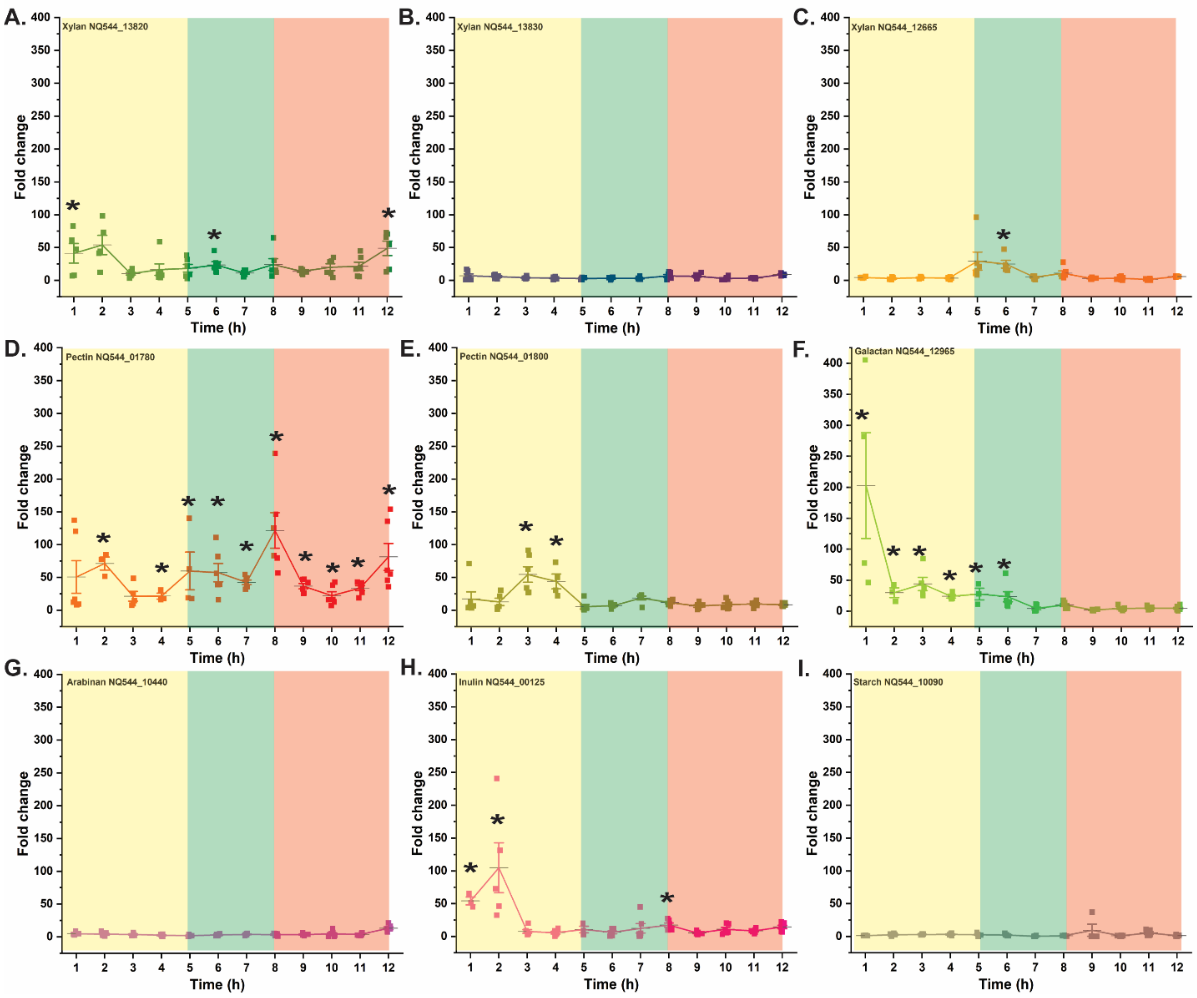

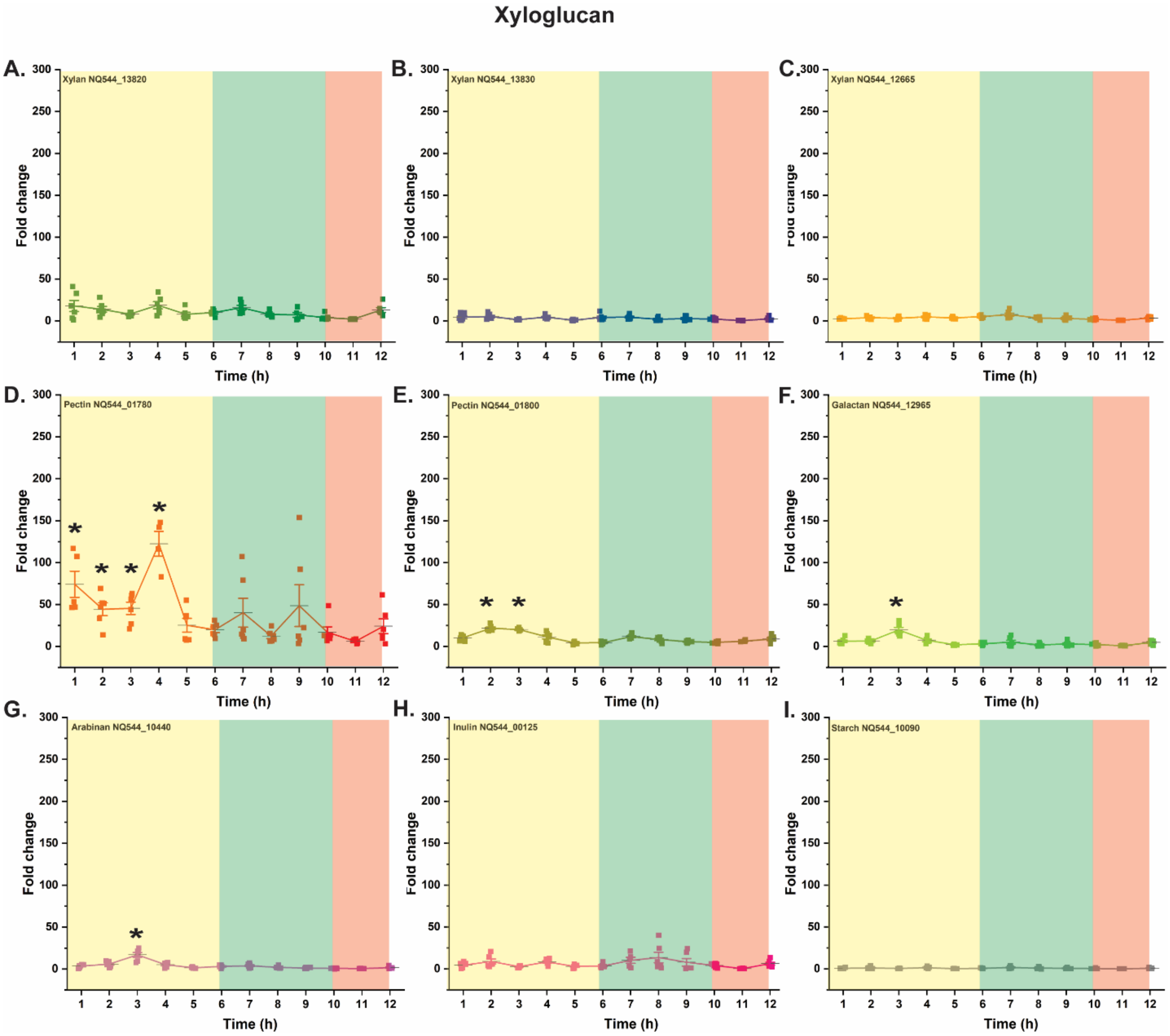

Figure S5. Temporal expression of xylan, pectin, and storage polysaccharide-associated *tbdt* genes during growth of *Sc*DSM18205 in mYCFA supplemented with mixed linkage β-glucan and xyloglucan as sole polysaccharide sources. Upper panel mixed linkage glucan and lower panel xyloglucan **(A-C)** Xylan-associated *tbdt* genes **(D-G)** Pectin-associated *tbdt* genes. **(H and I)** Inulin- and starch-associated *tbdt* genes. Changes in transcript levels at each time point were relative to time-zero, before exposure to the polysaccharide. Error bar represents the SEM of three technical replicates from single biological sample. Asterisks indicate data points with upregulation significantly greater than a basal level (set at 10-fold), as determined by an unpaired *t* test.

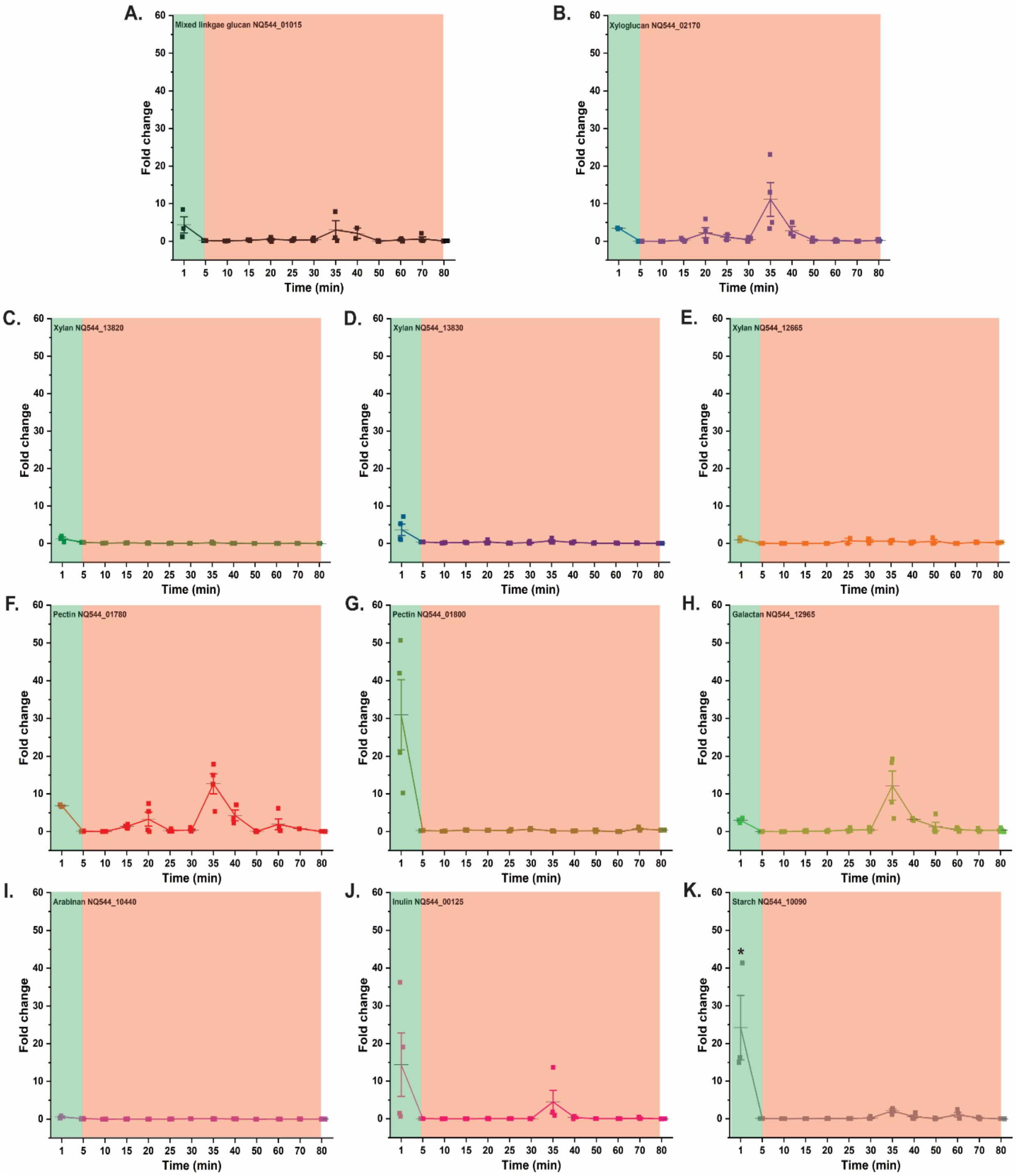

Figure S6. Temporal expression of hemicellulose, pectin, and storage polysaccharide-associated *tbdt* genes during growth of *Sc*DSM18205 in mYCFA supplemented with glucose as the sole carbohydrate source. Changes in transcript levels at each time point were relative to time-zero, before the inoculation of cells in the medium. Error bars represent the SEM of two biological replicates, and two technical replicates. Asterisks indicate data points with upregulation significantly greater than a basal level (set at 10-fold), as determined by an unpaired *t* test. *t* test.

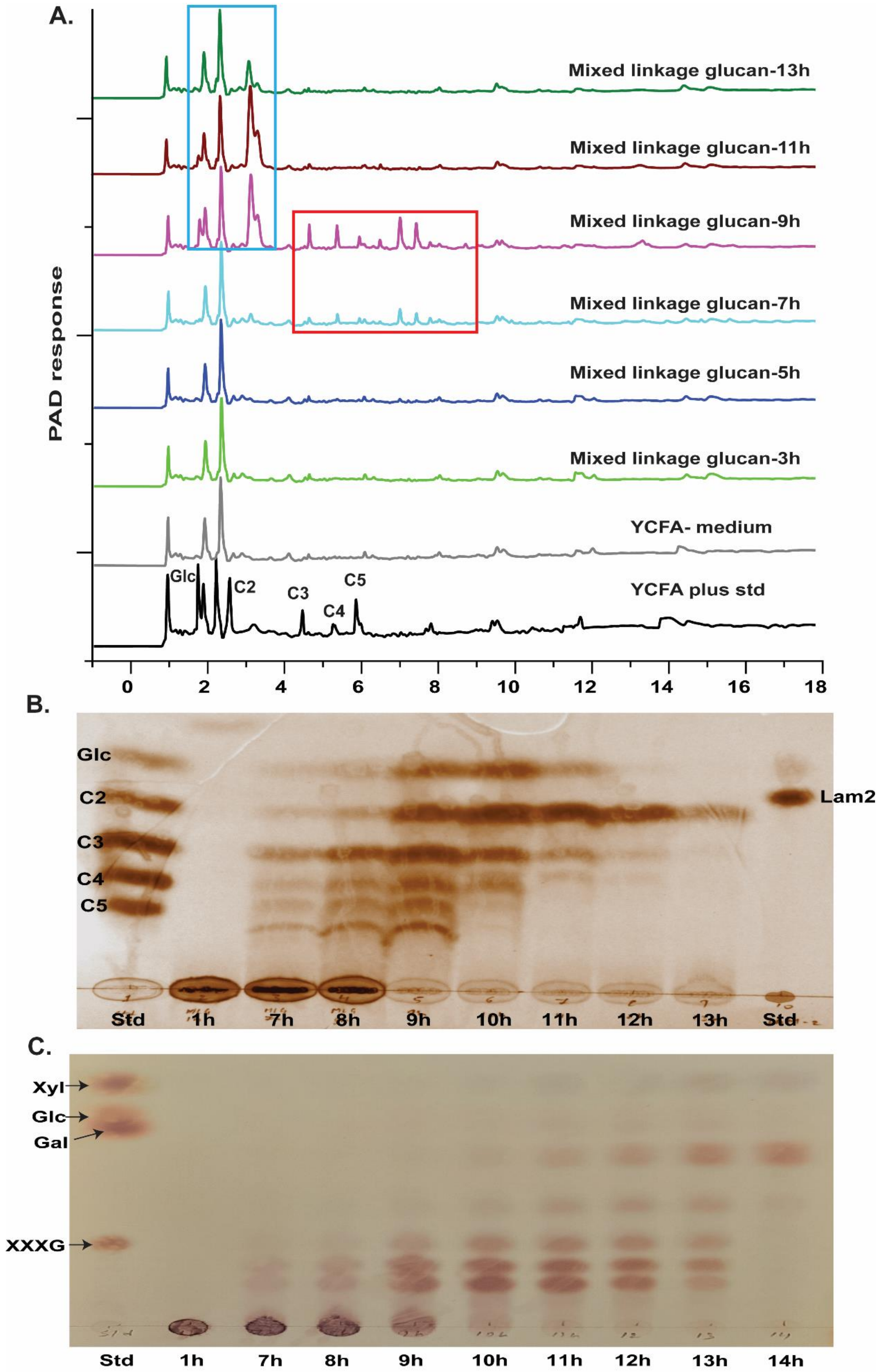
A.
PAD response
Mixed linkage glucan-13h
Mixed linkage glucan-11h
Mixed linkage glucan-9h
Mixed linkage glucan-7h
Mixed linkage glucan-5h
Mixed linkage glucan-3h
YCFA- medium
YCFA plus std
Glc
C2
C3
C4
C5
0
2
4
6
8
10
12
14
16
18
B.
Glc
C2
C3
C4
C5
Lam2
Std
1h
7h
8h
9h
10h
11h
12h
13h
Std
C.
Xyl
Glc
Gal
XXXG
Std
1h
7h
8h
9h
10h
11h
12h
13h
14h

Figure S7. Oligosaccharide production by *S. copri* DSM18205 during growth on mixed linkage β-glucan or xyloglucan. (A) HPAEC-PAD analysis of the medium during growth in mYCFA containing mixed β-linkage glucan. The red box highlights oligosaccharide accumulation, which are ultimately reduced to cellobiose and glucose (blue box). **(B)** TLC of analysis of the same samples as in (A), indicating the existence of longer polysaccharides at early time points (baseline spot), release of oligosaccharides and glucose into the medium, eventual consumption of glucose and oligosaccharides, and persistence of cellobiose. **(D)** TLC of the medium during growth in mYCFA containing xyloglucan. In all panels, "std" indicates the standards used in individual analyses: glucose (Glc), cellobiose (C2), cellotriose (C3), cellotetraose (C4), cellopentaose (C5), laminaribiose (Lam2), Xyl_3Glc_4 xyloglucan heptasaccharide (XXXG), xylose (Xyl), galactose (Gal).

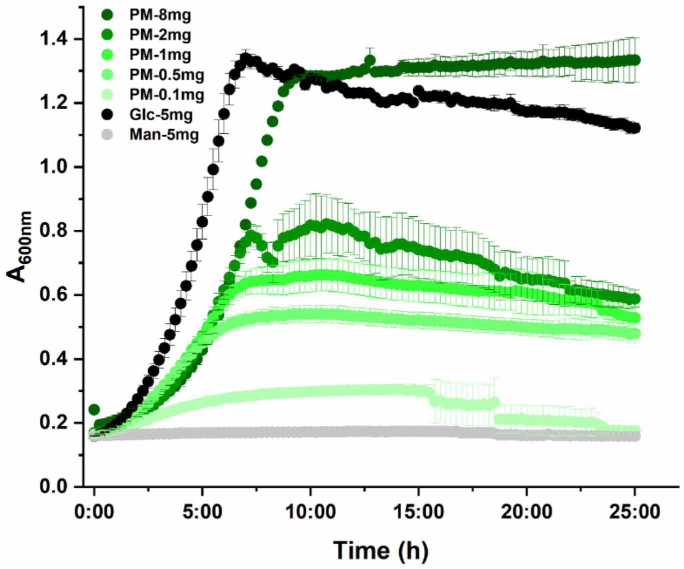

Figure S8. Growth kinetics of *Sc*DSM18205 in mYCFA supplemented with the polysaccharide mixture. (A) Growth kinetics were performed at different polysaccharide mixture concentrations (0.1 to 8 g/L) to examine carbon limitation. Glucose and mannose were used as positive and negative controls, respectively. Data are reported as the mean of three independent biological replicates with standard deviations indicated by error bars.

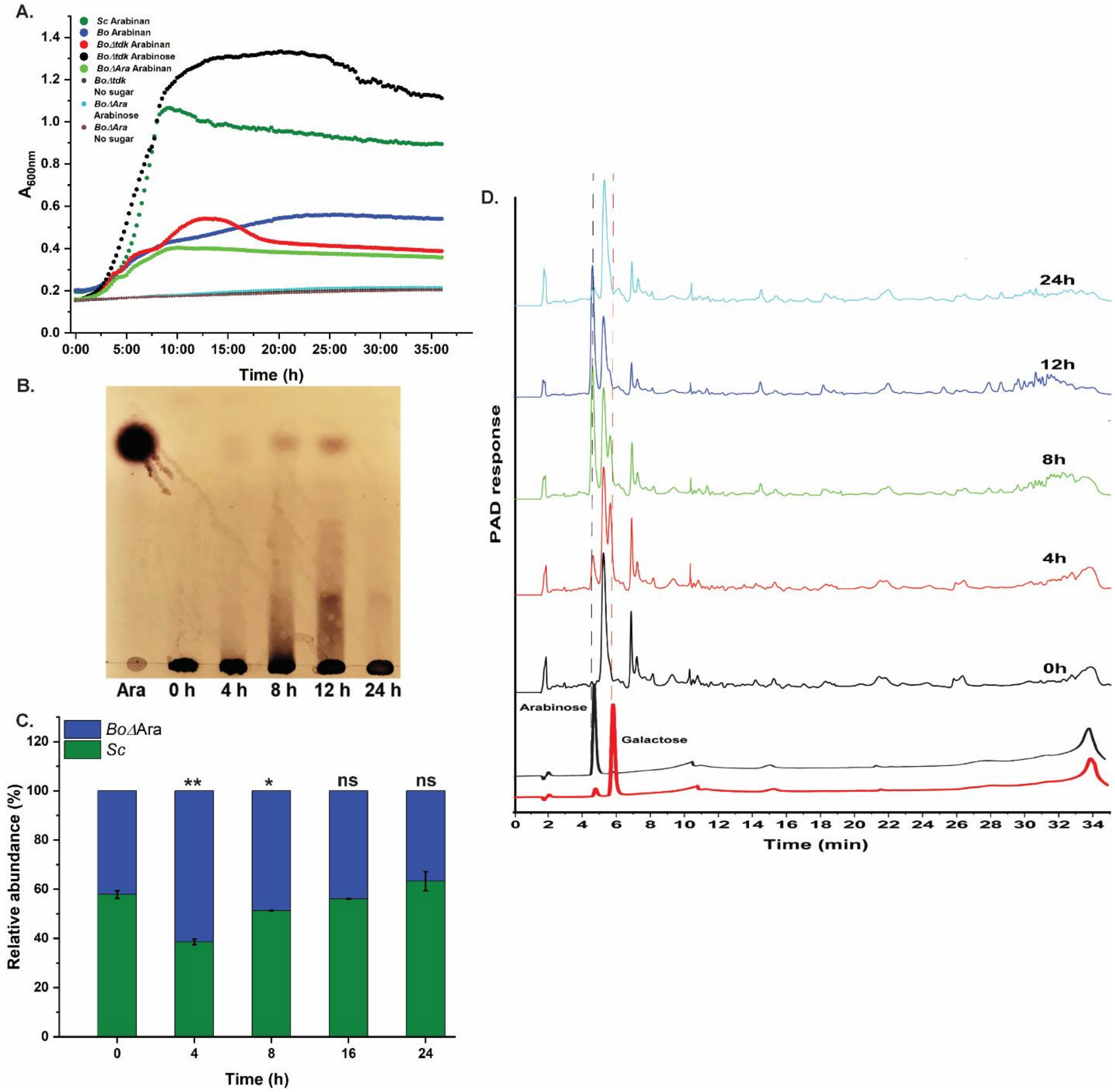

Figure S9. Utilization of pectic arabinan and galactan. (A) Growth of *Sc*DSM18205, *B. ovatus*, *B. ovatus Δtdk* (genetic background used for reverse genetics), and *B. ovatus ΔAra* (*Δtdk* with arabinose catabolism genes deleted) in mYCFA containing commercial pectic arabinan, arabinose, or no added carbohydrate source. **(B)** TLC showing accumulation of arabinose in the medium during co-culture of *Sc*DSM18205 and *B. ovatus* on arabinan. **(C)** Relative abundance of *Sc*DSM18205 and *B. ovatus ΔAra* during co-culture on arabinan. Statistically significant differences were calculated using a two-tailed unpaired Student's t test. *, $P < 0.05$; **, $P < 0.01$; ns, not significant ($P > 0.05$). **(D)** HPAEC-PAD chromatogram showing accumulation of both arabinose and galactose (from contaminating pectic galactan) in the medium during co-culture of *Sc*DSM18205 and *B. ovatus* on commercial arabinan.
